# Multiple Multilocus DNA Barcodes from the Plastid Genome Discriminate Plant Species Equally Well

**DOI:** 10.1371/journal.pone.0002802

**Published:** 2008-07-30

**Authors:** Aron J. Fazekas, Kevin S. Burgess, Prasad R. Kesanakurti, Sean W. Graham, Steven G. Newmaster, Brian C. Husband, Diana M. Percy, Mehrdad Hajibabaei, Spencer C. H. Barrett

**Affiliations:** 1 Department of Integrative Biology, University of Guelph, Guelph, Ontario, Canada; 2 Department of Ecology and Evolutionary Biology, University of Toronto, Toronto, Ontario, Canada; 3 UBC Botanical Garden amd Centre for Plant Research, and Department of Botany, University of British Columbia, Vancouver, British Columbia, Canada; 4 Biodiversity Institute of Ontario, Department of Integrative Biology, University of Guelph, Guelph, Ontario, Canada; American Museum of Natural History, United States of America

## Abstract

A universal barcode system for land plants would be a valuable resource, with potential utility in fields as diverse as ecology, floristics, law enforcement and industry. However, the application of plant barcoding has been constrained by a lack of consensus regarding the most variable and technically practical DNA region(s). We compared eight candidate plant barcoding regions from the plastome and one from the mitochondrial genome for how well they discriminated the monophyly of 92 species in 32 diverse genera of land plants (*N* = 251 samples). The plastid markers comprise portions of five coding (*rpoB*, *rpoC1*, *rbcL*, *matK* and 23S rDNA) and three non-coding (*trnH-psbA*, *atpF–atpH*, and *psbK–psbI*) loci. Our survey included several taxonomically complex groups, and in all cases we examined multiple populations and species. The regions differed in their ability to discriminate species, and in ease of retrieval, in terms of amplification and sequencing success. Single locus resolution ranged from 7% (23S rDNA) to 59% (*trnH-psbA*) of species with well-supported monophyly. Sequence recovery rates were related primarily to amplification success (85–100% for plastid loci), with *mat*K requiring the greatest effort to achieve reasonable recovery (88% using 10 primer pairs). Several loci (*matK*, *psbK–psbI*, *trnH-psbA*) were problematic for generating fully bidirectional sequences. Setting aside technical issues related to amplification and sequencing, combining the more variable plastid markers provided clear benefits for resolving species, although with diminishing returns, as all combinations assessed using four to seven regions had only marginally different success rates (69–71%; values that were approached by several two- and three-region combinations). This performance plateau may indicate fundamental upper limits on the precision of species discrimination that is possible with DNA barcoding systems that include moderate numbers of plastid markers. Resolution to the contentious debate on plant barcoding should therefore involve increased attention to practical issues related to the ease of sequence recovery, global alignability, and marker redundancy in multilocus plant DNA barcoding systems.

## Introduction

The development of DNA barcoding markers for discriminating among the >300,000 species of land plants is an unresolved problem, in contrast to some other groups of organisms where effective barcoding markers are now available. A portion of the mitochondrial cytochrome c oxidase 1 (COI or *cox1*) gene sequence is currently being used as a universal barcode in certain animal groups [1], [2], but mitochondrial genes are generally thought to lack promise for plants [3]–[6], primarily because of their low nucleotide substitution rates (e.g., [7]). It is generally agreed that a multilocus approach based on plastid (‘chloroplast’) data is currently the most effective strategy for species identification and species recognition in plants [3]–[6], [8]–[10] (but see [11]). However, both the identity and number of the most appropriate regions for plant barcoding remain contentious (e.g., the competing proposals reviewed in [12]).

Plant researchers have proposed several different barcode regions. These focus on coding and non-coding regions located primarily in the plastid genome. Kress et al. [4] suggested that two non-coding regions (the nuclear ITS region and the plastid *trnH-psbA* intergenic spacer) may have potential as universal plant barcodes, but subsequently proposed the combination *rbcL* and *trnH-psbA* [8]. Other combinations involving three plastid regions have also been proposed by a working group that includes the Royal Botanical Gardens, Kew, UK (http://www.rbgkew.org.uk/barcoding). These comprise the *trnH-psbA* region and portions of two coding regions (*matK* and *rpoC1*), or three coding regions combined (*matK*, *rpoB* and *rpoC1*) [9]. Other regions, such as a portion of the plastid 23S rDNA locus (referred to as the “universal plastid amplicon” or UPA [13]), and the plastid *trnL–trnF* intergenic spacer [14] have been proposed. Additionally, it has been suggested that the mitochondrial *cox1* locus not be discounted as a plant barcode marker without sufficient evidence (P.D.N. Hebert, University of Guelph, pers. comm.). Based on a study focused primarily on Orchidaceae, Lahaye et al. [11], recently proposed that *matK* be adopted “as a universal DNA barcode for flowering plants” (either alone or in combination with *trnH-psbA*), leaving its suitability for other plants largely unresolved. Finally, at a recent conference (*Second International Barcode of Life Conference*), the Plant Working Group of the Consortium for the Barcode of Life (PWG-CBOL) proposed additional combinations of non-coding and coding plastid regions (i.e., *matK*+*atpF–atpH*+*trnH-psbA*; *matK*+*atpF–atpH*+*psbK–psbI*; see [12]).

Because of the size of the task in establishing a reference barcoding database for all plants, it is widely accepted that a single (“universal”) set of barcode regions should be adopted. To date there has been no comprehensive empirical test of the utility of all gene regions and multilocus combinations currently under consideration. The assessment of different solutions is complicated by the multiplicity of ideas on what the most relevant criteria are for choosing among different single and multilocus barcoding strategies, and by lack of clarity about the weightings that ought to be placed on their relative importance. The amount of variation obtained per sequencing read is obviously a key parameter (the more the better, within limits). However, other technical considerations are likely to be at least as important, such as the ease of sequence recovery, the frequency of DNA sequencing artifacts, and the question of whether DNA sequence alignment is useful for identifying species using barcodes. The Barcode of Life Data System (BOLD, www.boldsystems.org [15]) currently relies on aligned sequences, but other alignment-independent algorithms have been proposed or are in development (e.g., [16], [17]).

Here, we compare the ability of nine different gene regions to discriminate among species within clades of closely related organisms — portions of five coding plastid regions (*rbcL*, *matK*, *rpoC1*, *rpoB* and 23S rDNA), three non-coding plastid intergenic spacer regions (*trnH-psbA*, *atpF–atpH* and *psbK–psbI*), and the mitochondrial *cox1* gene (the animal barcode region). We tested these regions individually and in various combinations (including the most commonly proposed ones) for a diverse set of genera from major clades of land plants from temperate N. America. The surveyed genera include bryophytes, a lycophyte, and several monilophytes and gymnosperms, in addition to numerous angiosperms. Each species is represented by accessions from multiple populations.

We apply a distinct approach to evaluating species resolution here, by assessing whether clusters of populations that correspond to monophyletic species in a floristic sample are well supported (see [11] for a somewhat similar approach). A high degree of correlation should exist between support for species monophyly and the ability of DNA barcoding marker systems to discriminate species, since the robustness of the branch that subtends a single species (representing a monophyletic cluster of populations) determines whether that species is distinct from closely related species, at least within the limits of sampling error. Our survey necessarily focuses on gene-tree monophyly, because this is what is quantifiable using markers belonging to a single linkage group (e.g., the plastid genome). However, in a few cases we find evidence of gene-tree paraphyly that also raises questions about the monophyly of some sampled species. It is worth emphasizing that species assessed to have well-supported monophyly in a local context may not be monophyletic on a global scale (and in fact our locally focused sampling precludes testing this). Nonetheless, we argue that the strength of support for species monophyly in a local context provides a useful comparative framework (benchmark) for predicting the relative DNA barcoding success of different regions, or combinations of them in a larger sample. In addition, it provides an estimate of the upper bound to DNA barcoding success, since broader geographic sampling will only find more evidence against monophyly where such evidence exists.

## Results

In total, we obtained 2048 sequences from 251 samples (mean = 8.3 regions sequenced per sample) for the nine regions. Sequencing success averaged 91.9% overall, ranging from 72.0% for *cox1* to 100% for *rbcL* and 23S rDNA (Table 1). The nine regions differed in the degree of technical complexity that was required to retrieve them. Success primarily depended on whether amplification product could be obtained using available primers. Non-seed plants (bryophytes, lycophytes and monilophytes) were generally the most difficult to amplify, particularly *cox1* (where attempted), *matK* and *psbK–psbI*. In contrast, *rbcL* and *trnH-psbA* were readily retrievable for these taxa (Tables 2, S1). Overall, *matK* required the most effort, needing a larger number of primers than other regions to complete the sequences presented (although 10 primers were required in total for this region, we did not assay the comparative success of each PCR primer pair). In comparison, sequence recovery for the three non-coding regions involved only one primer pair each: almost all samples were amplifiable for *trnH-psbA*, with ∼5–15% failure rates for *atpF–atpH* and *psbK–psbI*, respectively (Table 1; note that we did not attempt to design new primers for either region), and with a particular concentration of failures in bryophytes, monilophytes and lycophytes for *psbK–psbI* (Table 2). Two of three coding regions (*rpoC1*, *rpoB*) had intermediate levels (5–8%) of unsuccessful amplifications.

**Table 1:** Sequencing success and efficacy for six coding and three non-coding regions.

Region:	*cox1*	23S rDNA	*rpoB*	*rpoC1*	*rbcL*	*matK*	*trnH-psbA*	*atpF–atpH*	*psbK–psbI*
Aligned sequence length (bp)	656	363	590	487	607	946 †	200–760 ††	242–735 ††	260–673 ††
Unaligned length; mean (range), including end gaps	656 (-)	362.7 (359–363)	470.5 (429–481)	487 (-)	606.8 (588–607)	735.3 (325–895)	392.7 (142–699)	545.5 (240–589)	403.0 (172–629)
Position in *Arabidopsis thaliana* gene (length) ‡	42–697 (656)	2091–2453 (363)	1704–2175 (472)	1895–2381 (487)	27–633 (607)	525–1309 (785)			
No. of species successfully amplified and sequenced *	69	90	87	89	92	84	92	88	79
No. of samples successfully amplified and sequenced **	170	236	231	238	251	220	249	239	214
% sequencing success ***	72.0	100.0	92.0	94.8	100	87.6	99.2	95.2	85.3
Total no. primer pairs used	1	1	5	3	2	10	1	1	1
Mean number of reads in contig per sample¶	2.00	2.00	2.27	2.48	2.27	2.96	2.83	2.44	2.67
% of sequences that are <80% bidirectional §	1.1	0	4.7	6.9	2.4	25.5	19.7	6.3	27.1

Sequences from the first seven regions were sought for 251 samples representing 92 species. *Cox1* and 23S rDNA were attempted for 236 samples and 90 species. The sequence ranges used in the analysis are provided in reference to the complete plastid and mitochondrial genomes of *Arabidopsis thaliana* (Genbank accessions NC 000932, NC 001284).

† Aligned across angiosperms and gymnosperms only.

†† Aligned across individual genera only.

‡ Based on trimmed alignments for coding regions.

* 92 species attempted, except 23S rDNA and *cox1* (90 species).

** 251 individuals attempted for all genes except 23S rDNA and *cox1* (236 individuals);

*** Percentage sequencing success (i.e., number of individuals successfully sequenced/number of individuals attempted);

¶ The number of reads represents the mean number of unidirectional sequences from successful amplifications that are required to establish a reliable sequence for each sample;

§ Sequences with less than 80% bidirectional coverage are primarily due to the presence of homopolymer runs.

**Table 2:** Number of species per genus resolved as monophyletic for each of nine candidate barcoding regions.

Major clade	Genus	No. of species surveyed	Region								
			*cox1*	23S rDNA	*rpoB*	*rpoC1*	*rbcL*	*matK*	*trnH-psbA*	*atpF–atpH*	*psbK–psbI*
Angiosperms	*Acer*	5	0	0	0	0	2	3	3	0	0
	*Betula*	2	0	0	0	0	0	0	0	M	0
	*Cornus*	4	0	0	1	0	1	2	2	1	3
	*Erigeron*	2	0	0	0	0	0	0	0	0	0
	*Eupatorium*	2	0	0	2	0	2	2	2	2	2
	*Lactuca*	2	NA	NA	2	2	2	2	2	2	2
	*Plantago*	3	3	1	1	1	1	3	2	1	1
	*Poa*	2	2	2	2	2	2	2	2	2	2
	*Polygonum*	4	0	0	2	1	3	3	3	3	3
	*Populus*	4	0	0	0	0	1	0	2	0	1
	*Quercus*	3	0	0	1	1	0	1	1	0	0
	*Rhamnus*	3	0	0	2	1	2	3	3	3	2
	*Rubus*	4	1	0	3	1	3	4	2	4	2
	*Salix*	3	0	0	1	0	1	1	2	1	2
	*Silene*	2	M	0	2	0	0	2	2	2	2
	*Solanum*	2	0	0	2	2	2	2	2	2	2
	*Solidago*	6	0	0	0	0	0	0	0	1	0
	*Sonchus*	2	NA	NA	M	0	0	M	0	0	0
	*Symphyotrichum*	7	0	0	0	0	2	1	0	0	0
	*Trifolium*	2	M	0	2	2	2	2	2	2	2
	*Typha*	2	0	0	0	0	0	0	0	0	0
	*Viburnum*	3	0	0	1	0	2	1	3	1	1
Gymnosperms	*Juniperus*	2	M	0	2	2	0	2	2	0	2
	*Picea*	2	NA	NA	0	M	2	2	0	0	2
	*Pinus*	3	1	0	2	1	3	3	3	2	2
Monilophytes	*Dryopteris*	3	M	0	M	1	1	M	1	1	1
	*Equisetum*	2	M	0	M	M	2	M	2	M	M
Lycophytes	*Lycopodium*	2	M	2	2	M	2	2	2	2	M
Bryophytes	*Brachythecium*	3	M	1	2	2	2	1†	3	3	M
	*Dicranum*	2	NA	NA	2	2	2	M	2	2	M
	*Plagiomnium*	2	NA	NA	M	2	2	M	2	2	M
	*Polytrichum*	2	M	0	2	2	2	M	2	2	M
No. of species amplified for at least two populations			58	82	85	88	92	80	92	88	78
Percent species monophyletic (of those amplified and sequenced)		-	10	7	43	29	48	56	59	45	44
Percent species monophyletic (of those attempted)		-	6.7	6.7	39.1	27.2	47.8	47.8	58.7	43.5	35.9

Values indicate the number of species for each genus identified as monophyletic with at least 70% bootstrap support. ‘M’ indicates that none of the species in that genus had more than one sample, or that only one species was amplified; ‘NA’: amplifications not attempted in all species for this genus.

† Low resolution (1 of 3 species) attributed to partial amplification success in this genus, rather than failure to form a monophyletic group.

The total laboratory effort required for amplification is also affected by the number of sequencing reads required per region (summarized in Table 1). Considering only the successful amplifications, seven of the nine regions required more than two successful sequencing reactions to yield contigs with sufficiently high quality (only *cox1* and 23S rDNA did not require resequencing). Three regions (*matK*, *trnH-psbA* and *psbK–psbI*) required the most effort, up to 50% additional sequencing beyond the minimum needed to obtain two-fold coverage (2.67–2.96 reads per contig; Table 1). These regions also had the highest degree of unidirectional coverage (∼20–27% of samples with at least 20% unidirectional sequence; Table 1). When we experienced difficulty in obtaining bidirectional reads this was primarily due to the existence of homopolymer runs (e.g., sporadically for all non-coding regions, but also for *mat*K in some instances).

### Single region analysis

The percentage of species resolved as monophyletic was usually very poor for the mitochondrial *cox1* and plastid 23S rDNA regions (10% and 7%, respectively) considered alone; each of these two regions was consistently outperformed by other single regions (Table 2) (except for *cox1* in *Plantago*, a genus known to have elevated rates of mitochondrial nucleotide substitution [18]). Two coding regions, *rbcL* and *rpoB*, had similar overall rates of resolution to two of the non-coding regions, *atpF–atpH* and *psbK–psbI* (42–48%), but *matK* and *trnH-psbA* had the highest individual resolution rates overall (56% and 59%, respectively). Ignoring *cox1* and 23S rDNA, several taxa were consistently resolved by all regions, considered individually (*Plantago lanceolata*, *Polygonum convolvulus* and all of the sampled species of *Lactuca*, *Poa*, *Solanum* and *Trifolium*). Most species outside the angiosperms and gymnosperms were consistently resolved by each region when we were able to generate data. In contrast, species of *Betula*, *Erigeron*, *Solidago*, *Sonchus*, *Typha* and most species of *Symphyotrichum* were not resolved by any single region.

### Multiregion analysis

**Figure 1:**
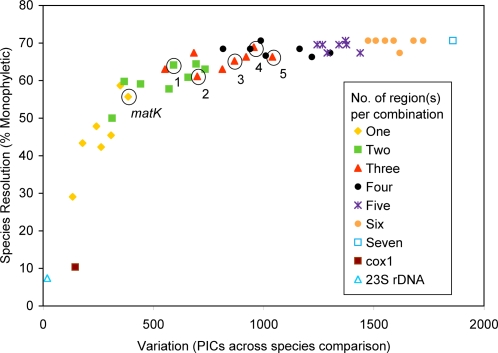
Relation between sequence variation (PICs = parsimony-informative characters summed across genus-level comparisons) and percentage species resolution (species supported as monophyletic within genera with at least 70% bootstrap support) for a selection of single and multilocus combinations. The number of regions used per combination is indicated by different symbols and colors (see legend). The specific regions used in each combination are noted in Table S2 (note that the combinations exclude *cox1* and 23S rDNA). Circled symbols correspond to combinations proposed in the recent plant barcoding literature (see text): 1) *rbcL*+*trnH-psbA*; 2) *matK*+*rpoC1*+*rpoB*; 3) *matK*+*rpoC1*+*trnH-psbA*; 4) *matK*+*atpF–atpH*+*psbK–psbI*; 5) *matK*+*atpF–atpH*+*trnH-psbA*. All regions are from the plastid genome (except *cox1*; mitochondrial genome).

**Figure 2:**
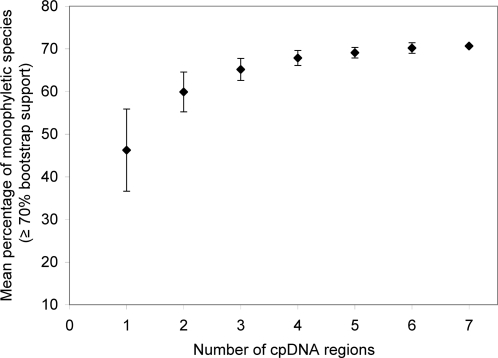
Relation between the number of plastid regions used and mean percentage species resolution (species supported as monophyletic within genera with at least 70% bootstrap support). Means (±SD) for two to six regions are based on the relatively arbitrarily chosen combinations of regions considered here (note that the plastid 23S rDNA locus and the mitochondrial locus *cox1* were not considered in these combinations). Least square regression: *R* ^2^ = 0.73; y = 0.52+0.11 ln x = 0.05 (ln x−1.1)^2^; *F*_1,43_ = 56.8, *P*<0.0001. Note: cpDNA = plastid DNA.

Combining regions improved the proportion of species resolved as monophyletic within genus-level clades (Figs. 1 and 2; note that *cox1* and 23S rDNA were not considered in these combinations). Species resolution was positively related to the amount of phylogenetically informative variation (parsimony informative characters, PICs, calculated within each genus and summed across them: Fig. 1; Table S2), and the number of regions used in combination (Fig. 2; Table S2). The variance among combinations decreased as more regions were combined, with the single, two and three-region combinations exhibiting the greatest variation (Fig. 2). Among two-region combinations examined, species resolution ranged from 50% with the *rpoB*+*rpoC1* combination, to 64% using *rbcL*+*trnH-psbA* or *matK*+*atpF–atpH*. Among three-region combinations examined, species resolution ranged from 61% to 69% (*matK*+*rpoB*+*rpoC1* had the lowest resolution; *matK*+*atpF–atpH*+*psbK–psbI* had the highest resolution). The success of species resolution reached an asymptote of 71% with increasing numbers of regions considered in each combination (Fig. 1). This plateau in performance was approached with four regions, with percentage species resolution barely changing with addition of further regions (Fig. 2).

The bootstrap values generated from a seed-plant-wide alignment of the four coding regions using the ‘fast bootstrap’ option in PAUP* [19], generally agreed well with values generated using this gene combination for each genus considered separately. Our species monophyly estimates were all obtained in the context of individual genera (see Materials and Methods), and so it is notable that all genera were supported as clades at the current limited taxon samplings in analysis of this seed-plant alignment (data not shown). We next repeated the main analyses (performed within each genus) using more vs. less conservative bootstrap cut-offs (80% and 60%, instead of 70%) for considering a species to be well supported, to see whether this affected the overall results. We found these different cut-offs had little effect on maximum resolution (data not shown). With a 60% threshold, the resolution of some single regions and combinations of two and three regions increased towards the maximum level of resolution obtained (71%). Using an 80% threshold resulted in a slower approach to the plateau in performance, but the resolution was nearly identical with a ∼70% threshold for all combinations of four or more regions.

We also re-evaluated the results described above by excluding all individuals with one or more missing regions (∼23% of samples had at least one of the seven regions missing: ∼8% had one region missing, ∼10% two regions missing, and less than 2% of samples had 3–5 regions missing in each case, respectively). We used ANOVA to evaluate the joint effects of deleting samples and the number of regions on species resolution. As might be expected, the number of regions used had a significant effect (*F*_6,76_ = 40.6, *P*<0.0001) on percentage species resolution, whereas deleting samples had no effect (*F*_1,76_ = 0; *P* = 1). An insignificant interaction indicated that missing samples here had no effects on the relation between region number and species resolution (*F*_6,76_ = 0.13, *P* = 0.99).

Overall there were 27 species in our dataset that could not be resolved even with the seven-region combination. Of these, four (15% of unresolved species) are in two genera (*Acer*, *Dryopteris*) that show variation in the plastid regions, but in which one or more species are clearly not monophyletic. The remaining species (85%) are in groups that exhibit very little (or no) variation in the plastid regions examined, with identical or nearly identical multilocus barcodes shared by samples from different species. These include the species of *Betula*, *Erigeron*, *Solidago*, *Sonchus*, *Typha* and *Symphyotrichum* that were not resolved by any single region.

Species resolution differed among the five plastid DNA combinations proposed in the literature (Table 3), although the range was not large. The highest species resolution was observed in the combination *matK*+*atpF–atpH*+*psbK–psbI* (proposed by Ki-Joong Kim, School of Life Sciences and Biotechnology, Korea University, Seoul, Korea; see [20]), in which 69% of species were resolved and the lowest resolution (61%) was obtained from the combination *rpoB*+*rpoC1*+*matK* (proposed by Chase et al. [9]). There was considerable overlap among the combination categories, both in terms of the mean resolution and the number of parsimony informative characters, with several combinations of three regions falling in the 65–70% range (Figs. 1, and 2).

**Table 3:** Number of species per genus resolved as monophyletic for each of five proposed multilocus barcoding combinations.

Citation for the proposed combination:			Proposed barcoding combinations				
			*rbcL*+*trnH-psbA*	*matK*+*rpoC1*+*rpoB*	*matK*+*rpoC1*+*trnH-psbA*	*matK*+*atpF–atpH*+*trnH-psbA*	*matK*+*atpF–atpH*+*psbK–psbI*
			Kress and Erickson [8]	Chase et al. [9]	Chase et al. [9]	Lee et al. [20]	Lee et al. [20]
Major clade	Genus	No. of species surveyed					
Angiosperms	*Acer*	5	3	3	3	3	3
	*Betula*	2	0	0	0	M	M
	*Cornus*	4	4	2	4	4	4
	*Erigeron*	2	0	0	0	0	0
	*Eupatorium*	2	2	2	2	2	2
	*Lactuca*	2	2	2	2	2	2
	*Plantago*	3	2	3	3	3	3
	*Poa*	2	2	2	2	2	2
	*Polygonum*	4	4	3	4	4	3
	*Populus*	4	3	1	2	2	3
	*Quercus*	3	1	1	1	1	1
	*Rhamnus*	3	3	3	3	3	3
	*Rubus*	4	3	4	4	4	4
	*Salix*	3	2	2	2	2	3
	*Silene*	2	2	2	2	2	2
	*Solanum*	2	2	2	2	2	2
	*Solidago*	6	0	0	0	0	0
	*Sonchus*	2	0	M	M	M	M
	*Symphyotrichum*	7	0	2	2	1	2
	*Trifolium*	2	2	2	2	2	2
	*Typha*	2	0	0	0	0	0
	*Viburnum*	3	3	2	3	3	3
Gymnosperms	*Juniperus*	2	2	2	2	2	2
	*Picea*	2	0	0	0	2	2
	*Pinus*	3	3	3	3	3	3
Monilophytes	*Dryopteris*	3	1	1	1	1	1
	*Equisetum*	2	2	M	0	0	M
Lycophytes	*Lycopodium*	2	2	2	2	2	2
Bryophytes	*Brachythecium*	3	3	3	3	3	3
	*Dicranum*	2	2	2	2	2	2
	*Plagiomnium*	2	2	2	2	2	2
	*Polytrichum*	2	2	2	2	2	2
No. of species amplified for at least two populations			92	90	92	92	90
Percent species monophyletic (of those amplified and sequenced)			64	61	65	66	69
Percent species monophyletic (of those attempted)			64	60	65	66	67

Values indicate the number of species for each genus identified as monophyletic with at least 70% bootstrap support for each multi-locus combination; ‘M’ indicates that none of the species in that genus had more than one sample, or that only one species was amplified and sequenced.

## Discussion

The search for a universal barcode for plants has generated intense debate within the botanical community, in addition to considerable public interest. Multiple solutions have been proposed in terms of the number and combination of barcoding regions, but no clear consensus has yet emerged (e.g., [12], [21]). To some extent, the difference of opinion is related to the criteria deemed most important for measuring barcoding success. A major factor in the failure to reach a consensus has been the lack of a satisfactory or standardized metric for assessing success in species assignment or discriminability [21]. We agree with Kress and Erickson [21] that a plant barcoding system should be effective for all land plants, not just angiosperms, to permit their use in floristic and ecological applications. We also distinguish between the technical difficulties associated with retrieving each region from the relative success of each region or multilocus combination in species discrimination. Some previous studies have tended to confound these when quantifying barcoding success [21], [22], although both are important. Most of our discussion focuses on seven core plastid regions, as we readily ruled out two of the regions as standard solutions to plant barcoding (*cox1*, 23S rDNA) due to their poor ability to resolve species monophyly (Table 1; Fig. 1).

### Ability to resolve monophyletic clusters that correspond to individual species

We used a metric that focuses on the monophyly of individual species; that is, the percentage of well-supported monophyletic species across our sample of taxa. Based on this criterion we demonstrate that no single barcoding region has an ability to resolve species to the same degree as almost any of the multilocus barcoding solutions we evaluated (Fig. 1, Table S2; note that a few two-gene combinations were poorer than *trnH-psbA* or *matK* alone)

A single-locus solution to plant barcoding is therefore substantially less effective than a multilocus system, and the variation in resolving power between most multilocus systems is marginal, at best, particularly when four or more loci are considered. The small differences that we observed at this level of combination are likely a function of the particular sampling of taxa we surveyed, although it is probable that a larger sampling of taxa would provide finer-scale comparisons of success rates. We examined all combinations of loci that were recently proposed at the *Second International Barcode of Life Conference* (see [12]). These all performed about as well as each other, ranging from a low of 61% (*matK*+*rpoC1*+*rpoB*) to a high of 69% (*matK*+*atpF–atpH*+*psbK–psbI*) (Fig. 1, Table 3), just below the maximum observed (71%). A number of additional combinations of two and three gene regions also fall into this range (Fig. 1, Table S2). When only one or two barcoding regions are considered, species resolution is moderately to strongly related to the number of parsimony informative characters. The strong but declining relationship between the number of regions used and the mean percentage of species resolved (Fig. 2) reinforces the point that species resolution is less dependent on the particular regions used than the number of regions. While the general shape of the curve is predicted by combining data in this manner, the observed reduction in variance with additional regions indicates that most of the multilocus combinations discriminate nearly the same number of species. All else being equal (i.e., setting aside technical differences related to the ease of retrieval of DNA barcodes from samples), it follows that the component regions in these multilocus combinations are largely interchangeable in any multilocus plastid-based DNA barcoding system that consists of more than two loci.

It could be argued that the plateau in species resolution and reduced variance that occurs with increasing numbers of loci may be due, in part, to the high number of regions shared among different multi-locus comparisons. We do not doubt that this is a contributing factor. However, comparable rates of resolution were seen when we considered independent combinations that shared no regions in common. For example, among those combinations assessed here (Table S2), the mean resolution for all independently drawn two-locus combinations (mean of three independent pairs drawn three different ways from the eight possible pairs; Table S2) was 61%, and resolution for all independent three-locus combinations (mean of two independent triplets drawn four different ways from the eight possible triplets; e.g. *rpoB+rpoCI+rbcL* and *trnH-psbA*+*atpF–atpH*+*psbK–psbI*; Table S2) was 65%. These values are comparable to the means of 60% and 65% for two and three locus combinations (Fig. 2), which include some lack of independence. While it is possible that some unexamined two- or three-region combinations might work better than those considered, an exhaustive comparison of all possible combinations is unlikely to yield combinations that will be more successful across all land plants.

Species resolution is likely limited both by lack of resolving power for monophyly (reflecting rapid successive speciation events) and by ambiguities in species boundaries (a consequence of various “paraphyly” issues; see below). Unexamined regions of the plastid genome are unlikely to resolve the first problem, as the current regions were chosen for their relatively rapid rate of evolution [6] and they will all be similarly affected by phenomena like failed coalescence and introgression, wherever these may occur. Although species resolution and the number of informative characters are well correlated, the best single gene regions did not necessarily perform the best in combination. For example, the two best single gene resolution rates were attained with *matK* and *trnH-psbA* (56% and 59%, respectively), however both *rbcL*+*trnH-psbA*, and *matK*+*atpF–atpH* had marginally better resolution than *matK*+*trnH-psbA* (both 64%, versus 63%). Examination of the particular species resolved by these combinations reveals that not all regions are complementary. Certain species are resolved only by inclusion of differing sets of specific regions; even the worst performing region (*rpoC1*) provided informative characters for some species for which the best performing region (*trnH-psbA*) did not. This suggests that regardless of the region(s) ultimately adopted for plant barcoding, there will always be some species that would be better resolved by some other region. The success of a given region or combination is therefore likely to be in part an idiosyncratic function of the set of taxa surveyed.

### Predicting barcoding success based on the monophyly of species

We assessed the various proposals for plant DNA barcoding using the percentage of well-supported monophyletic species across our sample of taxa as a criterion for predicting successful species resolution. Does this constitute a rigorous method for comparing gene regions? The relation between bootstrap support for species monophyly in a gene tree and barcoding discrimination should be tightly linked: clearly, when a species is not confidently distinct from related species, new sequences may not be reliably assignable to the “correct” species. In effect, bootstrapping provides a measure of the expected mis-assignment due to local homoplasy, or of assignment failure due to simple lack of evidence of species monophyly. It measures the strength of support (“confidence”) for the species branch: If there is no clear evidence for this, a confident assignment may not be possible by any barcoding assignment method, unless, perhaps, one is certain that all relevant haplotypes have been sampled (although see [42]). It follows that “consistent non-zero sequence variation that distinguishes two species” [21] may not practically distinguish closely related species if one or more of these species is nested in another ( = non-monophyly), or if there is at least the possibility that they are. Our results also suggest that the branch separating two species may often be too short to detect with reasonable quantities of plastid data (29% of species not resolved, even with seven loci combined). Few would currently accept that a system with more than seven loci would be suitable as a universal plant barcode.

There are several possible complications with using bootstrap support for species monophyly as a metric for measuring barcoding success. First, not all species may be mutually monophyletic with their closest relatives. For example, one species may in practice be nested within another, making the latter paraphyletic at the species level (e.g., speciation of peripheral isolates, and the plesiospecies-apospecies concept of Olmstead [23]). Even when species are mutually monophyletic across much of their genomic complement, “mis-assignment” of individuals to the “wrong” species cluster for a given barcode (single or multi-locus) may reflect coalescence failure, introgression of the particular linkage group under study, or repeated origins of polyploid species (e.g., [24]). We noted any strongly supported paraphyly when we encountered it. However, these various “paraphyly” phenomena, reflecting the often complex genetics of plant species, should affect all plastid loci equally, as this genome is a single genetic linkage group. Because these phenomena should act consistently across plastid loci in a given sample of plants, they can be effectively ignored in our comparisons of the relative efficacy of different plastid regions or combinations in resolving monophyly.

Second, a related problem is that the bootstrap support criterion measures monophyly only in the context of the surveyed samples, and necessarily ignores the possibility that unsampled individuals from the same or different species might have disrupted monophyly had they been sampled (e.g., due to unsampled paraphyly, coalescence failures, or successful introgression events). Indeed, paraphyly, broadly construed, is thought to be a widespread phenomenon in plants [25], [26]. Because of this, the maximum set of species we resolved as monophyletic undoubtedly includes false positives, but this should affect all current barcoding methods that use comparable loci and taxon sampling. Current assignment methods may implicitly assume reciprocal monophyly of closely related species (e.g., comparison of within vs. between species variation); here, we noted only four species that approach well-supported paraphyly (*Acer rubrum*, *A. saccharinum*, *Dryopteris carthusiana*, *D. intermedia*). The geographic taxon sampling strategy we employed might generally be expected to upwardly bias the support for monophyly, since it does not sample all of the biological complexity of each species and all its relatives across their range. We attempted to mitigate this by examining multiple sets of closely related species, where possible, and by including representatives from at least two geographically disjunct populations. Within the limits of our local floristic sampling, we can only partly control for the bias, but again, this should affect all plastid regions equally, given their location on the same linkage group.

Third, all the assessments we made were in analyses that focused on individual genera, because it was not possible to unambiguously align non-coding regions beyond the genus level. The actual placement of the relevant root branch for a genus ( = local clade at the current taxon sampling) was therefore untested in these unrooted analyses by design, and may in reality sometimes fall inside one of the clusters corresponding to individual species. This would also have the effect of biasing our support values upwards, since we may have recognized some between-species splits that are not real. However, in general where we observed well supported taxon splits within genera, these also corresponded to monophyletic species in a combined analysis of a seed-plant alignment of the four coding regions (see Results), suggesting that this source of bias, if it occurs here at all, may be minor. This effect should be consistent across plastid data sets, again a function of their genetic linkage.

Finally, we do not necessarily intend the species-monophyly support measure to be a general assignment tool in barcoding applications, as bootstrapping and related approaches (e.g., jackknifing) are computationally demanding, although bootstrapping without branch swapping (“fast bootstrapping”) can be performed relatively rapidly for large alignments, and provides a conservative estimate of branch support [27]. More rapid assignment criteria such as those used in BOLD may be preferred in barcoding applications. However, we use species monophyly here as a metric for comparing the efficacy of different regions in the context of our very incomplete species and population sampling. We note that it is estimated that there are 320,000–400,000 species of seed plants alone [28], [29], and only a small fraction of these have been examined to date for barcoding markers.

### Paraphyly and limits to plant barcoding

One of the strengths of our study is the consistent inclusion of multiple samples for species, and multiple species per genus. These are critical components of any evaluation of potential barcoding regions, which have often been lacking in previous studies. While failure is usually ascribed to lack of variation between species, this is only one possible explanation. In this data set, we estimate that 4 of 27 (15%) of the total monophyly failures are not due to lack of detected variation, but rather to “paraphyly” (of whatever source) in the trees examined. The failures due to paraphyly include two species of *Acer* (*A. rubrum*, *A. saccharinum*), and two species of *Dryopteris* (*D. carthusiana*, *D. intermedia*). The paraphyly in *Dryopteris* is particularly well supported (e.g., 96–100% with three or more loci; data not shown), but paraphyly of two species in *Acer* is supported by only three parsimony informative characters when all seven regions are included (bootstrap support 75–86%).

Although we compared the largest number of congeneric species and regions to date, our absolute species number is still small in the context of an entire flora. It should be emphasized that as a result of our “thinly sampled” experimental design (relative to land-plant phylogeny as a whole), the maximum resolution that we report here should tend to be on the upper end of what is possible with a broader sampling of species and populations in the clades we considered. However, our maximum (71%) is lower than other values that have been reported in plant barcoding studies. Some of these studies may be biased as a result of having included too few close relatives. For example, although the recent study by Lahaye et al. [11] reports a 90% success rate for *matK*, they include a number of genera, and even some families, represented by only a single species (see [21]). The very large interspecific distances that result from this approach have the potential to inflate support for species monophyly (e.g., where two distant taxa are each other's closest relatives in a given survey). Conversely, we have not shied away from including congeneric species of taxonomically complex groups that are morphologically difficult to distinguish as a method of challenging the proposed regions. We estimate that exclusion of the two most intractable genera in our data set (*Solidago*, *Symphyotrichum*) would result in an increase in resolution of approximately 10% for all of the single gene regions and combinations. However, the inclusion of more populations per species or more species per genus would generally be expected to decrease it.

### Technical problems with the proposed regions

*MatK* is present in a number of previous barcoding proposals and has been suggested as sufficient for the task of barcoding plants [21]. In our study, *matK* provided among the highest species resolution of any single region; however, its success is complicated by the technical difficulties in retrieving sequences (Table 1). Despite the considerable effort that we made to retrieve it across all individuals (several sets of new primers, different sequencing chemistry) *matK* had a relatively low amplification success (88%), Lahaye et al. [11] reported that amplification in *matK* was straightforward with a single primer set. To test their claim of primer universality, we attempted to amplify all our samples with the primer set they used. We achieved <50% success in these comparisons. Similar difficulties have been reported by Kress and Erickson [8] and Sass et al. [22]. The Plant Working Group (http://www.kew.org/barcoding/update.html) and Ki-Joong Kim (pers. comm.) (Table S1) have put considerable effort into designing a number of primers targeting *matK* to address this problem. However, most of these have been designed for seed plants; as can be seen in Table 2, much of our missing data for *matK* is in the remaining land plants. With a limited amount of data available for *matK* in these groups, additional effort may be required for primer design. As our data collection phase coincided with development and publication of primers from other researchers, we did not perform comprehensive evaluations of the available primer sets. Nonetheless, it is fair to say that no universal (or nearly so) set of primers for amplifying *matK* currently exists.

We believe that a high percentage of bidirectional reads will be critical for a successful plant barcoding system, given the generally low amount of variation that separates many plant species [8], [30], and the increased danger of mis-assignment due to sequencing error that can be anticipated with incomplete bidirectional reads. Sporadic homopolymer runs regularly prevented us from obtaining fully bidirectional reads for two of the non-coding regions (*trnH-psbA* and *psbK–psbI*) and one of the coding regions (*matK*, mostly restricted to two genera); these three regions had by far the highest percentage of partly unidirectional reads (Table 1).

Maximum variation in the adopted barcode region(s) is clearly critical. From this perspective, non-coding regions are particularly important, as they tend to have the most variation (Table 1). However, they are also typically too difficult to align among distantly related organisms. The inclusion of highly variable non-coding regions generally precludes global sequence alignment across land plants, or even angiosperms. This introduces a new set of challenges, as the current Barcode of Life Data System (BOLD, www.boldsystems.org [15]) relies on distance measures generated from aligned sequences. The metric we used for predicting the assignment success of barcoding markers also requires alignments. An accurate alignment also facilitated the detection of short inversions in each of the non-coding regions examined. We noted small inversions in some sequences of the non-coding regions: Four inversions were seen in the *trnH-psbA* intergenic spacer (one each in the genera *Quercus* and *Brachythecium*, and two in *Pinus*); *Plantago*, *Salix* and *Juniperus* had one inversion each in *psbK–psbI*; *Solanum* had one inversion in *atpF–atpH*. While the inversions we noted were all species-limited for the current sample, their presence can be problematic [20]. Short inversions can re-invert with a high frequency (e.g., [31]), potentially yielding polymorphisms within one or more species. When inversions are not detected, this could lead to parallel inversion alleles from closely related species becoming artificially clustered together.

Our comparative survey focused on individual genera, because we were generally not comfortable aligning most of the non-coding regions beyond the level of genus. This contrasts with Lahaye et al. [11], who aligned one of the non-coding regions, *trnH-psbA*, from taxa across several orders. While this may be possible in a subset of cases, alignment of non-coding barcoding regions will be generally intractable across broader levels of phylogeny. Database support for non-coding regions may soon be provided, as BOLD is now providing support for ITS as a barcode for fungi. However, the debate on how to handle non-coding regions from a bioinformatics perspective is not settled (e.g., [9], [16], [21]). From the perspective of land plant alignments, *matK* is also problematic, as it was difficult to align seed plants with other land plants (e.g., the seed plant alignment took about two person days to complete and carefully check for the current small sample of taxa).

### Some recommendations

A universal plant barcode should include multiple regions. From the perspective of species resolution, the identity of the regions used is less important than the number. It should also be recognized that there are fundamental upper limits to what is possible for any current plant DNA barcoding approach. An ideal system should come close to these limits, and include markers that are straightforward to amplify, sequence and align. Unfortunately, none of the individual barcoding markers currently proposed simultaneously satisfy all these criteria (and it is unlikely that other single plastid markers exist that would). For example, in our study each coding region required at least two primer pairs (up to 10 for *matK*) and some (*matK*) required adjustment of PCR conditions; only *rbcL* was amplifiable in all individuals assessed here, and it was typically one of the least problematic for sequencing (but it and the other coding regions are considerably less variable than *matK* or the non-coding regions). *RpoB* and *rpoC1* were moderately more difficult to handle than *rbcL*; amplification failures were as high as 8% for *rpoB*, and *rpoC1* yields the fewest informative characters of the seven core regions we compared. Currently proposed non-coding regions have a variable rate of amplification from the single primer pairs we considered in each case (*trnH-psbA* was the best performing, with nearly 100% amplification; closely followed by *atpF–atpH*, with 4% failed amplifications). We experienced less sequencing and alignment problems for *atpF–atpH* than the other non-coding regions; i.e., it has a substantially higher frequency of bidirectional reads, fewer sequencing reads per amplification product, and (compared to *trnH-psbA*) fewer micro-inversions.

How, then, to proceed? Ignoring the technical issues related to DNA barcode retrieval, there are multiple multilocus plant DNA barcoding combinations that perform about equally well in resolving species. However, the debate about which combination of regions is “best” should now be focused more strongly on these sorts of practical issues. Based on our survey of nine candidate barcoding loci, we suggest that a multilocus plant barcoding region should have multiple regions chosen from among three of the coding (*rbcL*, *rpoB*, *matK*) and two of the non-coding regions (*trnH-psbA*, *atpF–atpH*). This should still be considered a flexible shortlist. For example, while *rpoC1* might be dropped from consideration due to its lower levels of variation, and *psbK*–*psbI* because of its higher failure rate in amplification and sequencing, these sorts of decisions boil down to the weights one is willing to place on different criteria for inclusion. If a heavy weight is placed on alignability (required for tree-based identification, which is recommended when sampling is sparse [42]), or the ability to produce fully bidirectional sequences, this would favor all of the coding regions except *matK*. If a high premium is placed on raw variation, then a multilocus barcode should include one or more non-coding regions. Critically, while we would suggest that *matK* continue to be considered given its high variability, this comes with the strong caveat that the significant technical issues for this locus, particularly relating to amplification (primer universality, especially, but not exclusively, for plants other than angiosperms), must be addressed in short order.

We demonstrate that three, and perhaps four regions yield close to optimal discriminating ability. Setting aside the technical challenges involved in obtaining and analyzing the regions it is clear that there are only marginal differences in the resolution possible with such combinations. Some may feel reluctant to consider more than two loci because of the greater costs involved. However, in our experience the main costs for barcoding relate to sample acquisition and processing (identification, DNA extraction), which are one-time costs that depend on the salaries of expert personnel. Indeed, as the costs of PCR and sequencing costs continue to decline rapidly relative to collecting expenses, the overall proportion of costs associated with them should become less of a concern. We therefore recommend that more regions than two should be preferred, because of the reduced variation in barcoding success in systems with three or more regions (Figs. 1 and 2), and because of the improved redundancy that this would provide when one or more regions cannot be recovered with satisfactory quality. Missing regions had no statistically significant effect here (see Results), but this could be misleading, as we made a substantially greater effort to reach a ‘complete’ sampling than might be feasible in real-world DNA barcoding applications (see Tables 1, S3). Additionally, if more conservative thresholds for support were favored, this would also tend to require the use of more loci. We hope that the practical issues related to our ability to deal with non-coding regions from a bioinformatics perspective can be satisfactorily resolved in the near future. If they cannot, a barcoding system with a higher proportion of readily alignable coding regions should be preferred, with little if any reduction expected in barcoding success. It is vital that the plant barcoding community adopt a consistent subset of regions as soon as possible to enable the assemblage of a global barcode database, permitting its application in floristic and ecological research.

## Materials and Methods

### Sampling

We sampled 92 species representing 32 genera primarily from locations in southern Ontario, Canada (Table S3). Our sampling over-represents some groups (gymnosperms, lycophytes) relative to the total number of land-plant species in these major clades (i.e., <1% in each case), and it is richest within the angiosperms, which are by far the largest group of land plants. In addition, although the number of true sister species pairs may be relatively low in the Ontario flora, our selection of taxa includes cases that are quite challenging for routine morphological identification, and which may also present a considerable challenge for barcoding (e.g., *Rubus*, *Salix*, *Solidago*, *Symphyotrichum*) due to hybridization (e.g., [32]–[34]), polyploidy (e.g., [34], [35]), and agamospermy (e.g., [35]). The selection of species for our study was based on a survey of the flora of the Koffler Scientific Reserve (KSR) at Jokers Hill near Newmarket, in Southern Ontario (44°03′N, 79°29′W). We selected genera that were each represented by at least two species, where we could sample each species from at least one other location outside the reserve (each location >80 km apart) (Table S3), giving a total of 251 individuals. The taxa we chose represent some of the most common species in the landscape of the region, including those most likely to be encountered during ecological/floristic applications of barcoding. The resulting complement of taxa contains sets of species that, based on our review of the literature and knowledge of the flora, exhibit potential for hybridization (e.g., *Acer*), polyploidy (e.g., *Dryopteris*), and phenotypic similarity (e.g., *Solidago*) that often make them difficult to tell apart using morphology. Specimens were mounted on herbarium sheets, photographed and stored at the University of Guelph Herbarium as barcode vouchers. For each specimen, we collected 3–5 cm^2^ of leaf tissue in the field stored in silica gel for DNA isolation.

### DNA isolation and amplification

For each sample, we isolated total genomic DNA from approximately 10 mg of dried leaf material using DNeasy 96 Plant Kits (QIAGEN) following manufacturer instructions. We performed DNA amplification with various annealing temperatures depending on the primers used (Table S1). In some instances the primer pairs available at the outset of the study did not work well for specific taxonomic groups. In particular, we designed new primers for both *rbcL*, and *cox1* and several sets of taxonomically-specific primer sets for *matK*, *rpoB* and *rpoC1* (Table S1). We did not test all the possible primer combinations on all samples, but rather designed primers for taxonomic groups as needed.

DNA was amplified in 20 ul reaction mixtures containing 1 U Ampli*Taq* Gold Polymerase with GeneAmp 10× PCR Buffer II (100 mM Tris-HCl pH 8.3, 500 mM KCl) and 2.5 mM MgCl_2_ (Applied Biosystems, Foster City, CA), 0.2 mM dNTPs, 0.1 µM of each primer (0.5 µM for *matK*), and ∼20 ng/ul template DNA. We sequenced amplification products directly in both directions with the primers used for amplification, following the protocols of the University of Guelph Genomics facility (http://www.uoguelph.ca/ib/facilities/Genomics/GenomicsFacility.shtml). For many of the *matK* amplification products, we encountered significant sequencing problems. The use of DMSO in the sequencing mix as suggested by Royal Botanic Gardens, Kew, UK [36] was helpful in some cases, but several samples required the use of dGTP BigDye Terminator (Applied Biosystems, Foster City, CA) sequencing mix. We cleaned sequence products from each specimen on Sephadex columns and ran the samples on an ABI 3730 sequencer (Applied Biosystems, Foster City, CA). We obtained bidirectional sequence reads from most PCR products, but in some cases either the forward or reverse sequencing reaction consistently failed, or only a partial sequence was recovered, frequently due to homopolymer runs. For these samples, a minimum of two-fold coverage was obtained by repeating the sequencing reactions in the direction that was successful initially, an approach that may, however, lead to more unrecognized sequencing errors than is possible with bidirectional reads. The mean number of reads performed per region, and the proportion of sequence with <80% bidirectional coverage is indicated in Table 1.

### Analysis

We assembled and base-called sequences using Sequencher 4.5 (Gene Codes Corp, Ann Arbor, MI), and aligned them manually using Bioedit version 7.0.9 [37] or Se-Al version 2.04 [38] following criteria laid out in Graham et al. [31]. For *rpoB*, *rpoC1*, *rbcL*, *cox1* and 23S rDNA it was possible to make global alignments with all samples. For *matK*, it was possible to align sequences of taxa across seed plants, but not outside them (alignments were then performed for each genus). Sequences from the non-coding regions (*trnH-psbA*, *atpF–atpH*, and *psbK–psbI*) were usually only readily alignable within genera, and so we did not attempt higher-order alignments for them. We therefore performed the main analyses (see below) separately for each genus

Four coding regions (*rpoB*, *rpoC1*, *rbcL* and *matK*) required the use of multiple primer sets for amplification. Consequently, the portion of these regions that was sequenced varied somewhat among taxonomic groups, depending on the primer set used. To minimize the amount of missing data in the resulting alignments, a small proportion of nucleotides at the 5′- and 3′-ends of the matrix for these four regions were excluded prior to analysis (to a point where at least 50% of sequences were full length on each edge). Because global alignments were not obtained for the non-coding regions, we simply included the entire sequence for each genus (ignoring incorporated primers, as usual). The nucleotide positions of the regions included in our analyses are provided in Table 1, relative to coordinates for corresponding genes in the plastid and mitochondrial genomes of *Arabidopsis thaliana*.

To estimate whether a species ought to be resolvable (discriminable) for a given genomic region or combination, we scored how well supported the monophyly of individual species was in bootstrap analysis of each genus (see also [11]). We used a reasonably conservative numerical cut-off (≥70%) (see [39], [40]) to define support for “successful” resolution as a monophyletic species. For a given region or combination we then determined the proportion of well-supported monophyletic species as a percentage of the total number of species. To determine whether using a different bootstrap threshold value would affect our overall results, we also calculated total resolution using more or less conservative (60% and 80%, respectively) thresholds

We performed bootstrap analyses on each genus [41] in PAUP* [19] using maximum parsimony, with MaxTrees set at 500, and with a single random addition replicate for each of 500 bootstrap replicates. We recorded the bootstrap values for each monophyletic species (or rather, taxon split in unrooted genus trees), and the number of parsimony informative characters per genus. For each gene region, we calculated identification success (species resolution) as the number of species forming monophyletic groups with bootstrap values 70% or greater, divided by the total number of species for which sequences were obtained (see Table 1).

The trees that we scored are unrooted and so genera that contain only two species were separated (if resolved) by a single branch, with a single bootstrap value. In these situations, we applied the bootstrap value to each species. To assess whether taxon splits on unrooted trees generally correspond with monophyletic species in rooted trees, we generated bootstrap values from a global alignment of the four coding regions for the seed plants (angiosperms and gymnosperms), and compared the profile of resolved species to what we saw when for each genus considered separately for the same four-region combination.

To assess the effect of multi-region combinations on species resolution, we selected a subset of the possible two-, three-, four-, five- and six-region combinations, in addition to the seven-region combination for analysis (Table S2) (*cox1* and 23S rDNA were excluded because of their low individual success, see Results). We chose specific combinations such that: 1) all five previously proposed barcode combinations [12] were included; 2) there were seven to eight different combinations for each multi-region set (two regions, three regions etc.); 3) the number of times that a given region was represented within each set was uniform, or nearly so; and 4) each set included the coding and non-coding combinations we expected to be least and most able to resolve species. In total, we analyzed 38 multi-region combinations in the same manner as the individual regions. We plotted species resolution for each of the resulting 47 comparisons (single regions and multi-region combinations) against the total number of parsimony-informative characters (PICs) summed across the genus-level analyses, which we used as a measure of the amount of information per region. We did not pro-rate the PICs by the sequence length in these comparisons (lengths of each region are variable, particularly for non-coding regions), since the most important outcome from a barcoding perspective is the amount of information retrieved for a region, not the amount of information per nucleotide sequenced. To determine the relation between number of regions used in the analysis and species resolution, we also performed a least-squares regression using JMP IN version 5.1.2 (SAS Institute Inc., SAS Campus Drive, Cary, NC.

To examine any potential bias in the estimates of total resolution due to missing data we repeated the analyses, excluding any samples that had data missing from any one of the regions of a multi-region set. Using JMP software (SAS Institute Inc., SAS Campus Drive, Cary, NC) we used ANOVA to evaluate the effects of method of analysis (species deleted versus not deleted), number of regions and their interaction on species resolution (JMP IN software, version 5.1.2, SAS Institute Inc. Cary, NC).

## Supporting Information

Table S1 Primer sequences and PCR conditions for eight plastid genomic regions and one mitochondrial region. PCR and sequencing reactions followed standard procedures described in the text; annealing temperatures varied among primers. See Table S3 for a complete list of species. ^1^Not all primer combinations were tested on all samples. Primers from: †[43]; ††[44]; ‡[36]; *This paper; **Ki-Joong Kim, School of Life Sciences and Biotechnology, Korea University, Seoul, Korea, unpublished primers kimkjkorea.ac.kr; ***[13]; ¶[4].
(0.08 MB DOC)

Table S2 Regions and combinations analyzed, with total number of parsimony informative characters (summed across genus-level comparisons) and species resolution (percentage of species supported as monophyletic with at least 70% bootstrap support). Single and combined regions are presented in order of increasing species resolution.
(0.10 MB DOC)

Table S3 GenBank and collection accession numbers for species sampled.
(0.65 MB DOC)
