# Supplementary material for: Multiple Multilocus DNA Barcodes from the Plastid Genome Discriminate Plant Species Equally Well
Source: PLoS One. 2008 Jul 30;3(7):e2802. doi: 10.1371/journal.pone.0002802 (PMC2475660; doi:10.1371/journal.pone.0002802)
Supplement: Table S1 — Primer sequences and PCR conditions for eight plastid genomic regions and one mitochondrial region. PCR and sequencing reactions followed standard procedures described in the text; annealing temperatures varied among primers. See Table S3 for a complete list of species. 1Not all primer combinations were tested on all samples. Primers from: †[43]; ††[44]; ‡[36]; *This paper; **Ki-Joong Kim, School of Life Sciences and Biotechnology, Korea University, Seoul, Korea, unpublished primers kimkjkorea.ac.kr; ***[13]; ¶[4]. (0.08 MB DOC) [file pone.0002802.s001.doc]

| Region | Primer combination | Successful primer sequence | Annealing temperature (°C) | Used in:^1^ |
| --- | --- | --- | --- | --- |
| *cox1* | **†**cox42F / ††cox1ajf699R | GGATCTTCTCCACTAACCACAA / CCGAAAGAGATGCTGGTATA | 55 | All except for bryophytes, monilophytes*, Lycopodium*, *Juniperus*, *Silene*, |
| *matK* | ‡matK 2.1af /  ‡matK 3r | ATCCATCTGGAAATCTTAGTTC / CTTCCTCTGTAAAGAATTC | *53-46* | *Rubus, Rhamnus, Quercus, Betula, Acer* |
|  | ‡matK 2.1af /  ‡matK 5r | ATCCATCTGGAAATCTTAGTTC / GTTCTAGCACAAGAAAGTCG | *53-46* | *Cornus, Symphyotrichum ericoides, Symphyotrichum lanceolatum, Symphyotrichum laterifolium* |
|  | ‡matK 2.1f /  ‡matK 5r | CCTATCCATCTGGAAATCTTAG / GTTCTAGCACAAGAAAGTCG | *53-46* | *Typha, Polygonum aviculare, Solanum, Sonchus* |
|  | ‡matK X F /  ‡matK 5r | TAATTTACGATCAATTCATTC / GTTCTAGCACAAGAAAGTCG | 45 | *Plantago* |
|  | *matKpkF1 /  *matKpkR1 | TTTCTGATGAACAARTGGAA / CGTATCGTGCTTTTRTGYTT | 46 | *Polygonum hydropiper, Polygonum persicaria, Symphyotrichum novae-angliae, Symphyotrichum pilosum, Symphyotrichum urophyllum, Erigeron, Solidago* |
|  | *matKpkF3 /  *matKpkR1 | CTACGATACTGGGTNAARGA / CGTATCGTGCTTTTRTGYTT | 48 | Viburnum |
|  | *matKpkF4 /  *matKpkR1 | CCCTATTCTATTCAYCCNGA / CGTATCGTGCTTTTRTGYTT | 50 | gymnosperms, *Lycopodium* |
|  | *matKpkF5 /  *matKpkR1 | CCTCATTTTATTCAYCCNGA / CGTATCGTGCTTTTRTGYTT | 50 | *Equisetum* |
|  | *matKpkF7 /  *matKpkR1 | CTAATACCCTACCCNATHCA / CGTATCGTGCTTTTRTGYTT | 46 | *Poa, Populus, Salix* |
|  | **3F_KIM /  **1R_KIM | CGTACAGTACTTTTGTGTTTACGAG / ACCCAGTCCATCTGGAAATCTTGGTTC | *58-53* | *Silene* |
| 23S rDNA | ***p23SrV F1 /  ***p23SrV R | GGACAGAAAGACCCTATGAAGCTT / TCAGCCTGTTATCCCTAGAGTAAC | 58 | All |
| *rbcL* | ¶rbcLa F /  ¶rbcLa R | ATGTCACCACAAACAGAGACTAAAGC / CTTCTGCTACAAATAAGAATCGATCTC | *58-53* | All except for *Poa, Lactuca, Eupatorium, Quercus, Typha, Betula* |
|  | ¶rbcLa F /  *rbcLajf634R | ATGTCACCACAAACAGAGACTAAAGC / GAAACGGTCTCTCCAACGCAT | *58-53* | *Acer, Betula, Cornus, Dicranum, Equisetum, Erigeron, Eupatorium, Lactuca, Picea, Pinus, Plagiomnium, Plantago, Poa, Polygonum, Populus, Quercus, Rhamnus, Rubus, Salix, Silene, Solanum, Solidago, Sonchus, Symphyotrichum, Trifolium, Typha, Viburnum* |
| *rpoB* | ‡rpoB 1f /  ‡rpoB 4r | AAGTGCATTGTTGGAACTGG / GATCCCAGCATCACAATTCC | *58-53* | All except for gymnosperms, bryophytes, monilophytes, *Lycopodium*, *Poa* |
|  | ‡rpoB 2f /  ‡rpoB 4r | ATGCAACGTCAAGCAGTTCC / GATCCCAGCATCACAATTCC | *58-53* | Poa |
|  | ‡rpoB 2f /  ‡rpoB LP5.2 R | ATGCAACGTCAAGCAGTTCC / AAATAAGGCATATCTTGTCT | *58-53* | gymnosperms |
|  | *rpoBajfF1 /  *rpoBajfR1 | TCTAATATGCAICGTCAAGC / GAGGIGTTAITTTACCTAC | *53-48* | bryophytes |
|  | ‡rpoB LP1.1 F /  ‡rpoB LP3 R | TCTAATATGCARCGTCAAGG / TTTACCCAAYRAAACATCHCC | *58-53* | monilophytes, *Lycopodium* |
| *rpoC1* | ‡rpoC1 1f /  ‡rpoC1 4r | GTGGATACACTTCTTGATAATGG / CCATAAGCATATCTTGAGTTGG | *58 – 53* | *Poa* |
|  | ‡rpoC1 2f /  ‡rpoC1 4r | GGCAAAGAGGGAAGATTTCG / CCATAAGCATATCTTGAGTTGG | *58 – 53* | All except for bryophytes, *Equisetum*, *Lycopodium* *obscurum, Poa,* |
|  | †rpoC1ajfMossF / †rpoC1ajfMossR | GGCAAAGAAGGACGTTTTCG / CCAGAAGCATATCTTGACTTGG | *58-53* | bryophytes |
| *trnH-psbA* | ¶trnH /  ¶psbA | CGCGCATGGTGGATTCACAATCC/ GTTATGCATGAACGTAATGCTC | *58-53* | All |
| *atpF-atpH* | **atpF /  **atpH | ACTCGCACACACTCCCTTTCC /  GCTTTTATGGAAGCTTTAACAAT | *58-53* | All except for *Betula*, *Equisetum* |
| *psbK-psbI* | **psbK /  **psbI | TTAGCCTTTGTTTGGCAAG /  AGAGTTTGAGAGTAAGCAT | *58-53* | All except for bryophytes, *Lycopodium, Equisetum* |
