# Supplementary material for: Multiple Multilocus DNA Barcodes from the Plastid Genome Discriminate Plant Species Equally Well
Source: PLoS One. 2008 Jul 30;3(7):e2802. doi: 10.1371/journal.pone.0002802 (PMC2475660; doi:10.1371/journal.pone.0002802)
Supplement: Table S2 — Regions and combinations analyzed, with total number of parsimony informative characters (summed across genus-level comparisons) and species resolution (percentage of species supported as monophyletic with at least 70% bootstrap support). Single and combined regions are presented in order of increasing species resolution. (0.10 MB DOC) [file pone.0002802.s002.doc]

| No. of regions | Combination | Total number of parsimony informative characters | Resolution (%) |
| --- | --- | --- | --- |
| 1 | 23S rDNA | 19 | 7 |
| 1 | *cox1* | 146 | 10 |
| 1 | *rpoC1* | 134 | 29 |
| 1 | *rpoB* | 179 | 43 |
| 1 | *psbK-psbI* | 263 | 44 |
| 1 | *atpF-atpH* | 308 | 45 |
| 1 | *rbcL* | 242 | 48 |
| 2 | *rpoB* + *rpoC1* | 313 | 50 |
| 1 | *matK* | 386 | 56 |
| 2 | *atpF-atpH* + *psbK-psbI* | 571 | 58 |
| 1 | *trnH-psbA* | 350 | 59 |
| 2 | *rpoB* + *psbK-psbI* | 442 | 59 |
| 2 | *rpoC1* + *rbcL* | 368 | 60 |
| 3 | *rpoB* + *rpoC1* + *matK* | 699 | 61 |
| 2 | *trnH-psbA* + *atpF-atpH* | 658 | 61 |
| 3 | *rbcL* + *atpF-atpH* + *psbK-psbI* | 813 | 63 |
| 3 | *rpoB* + *rpoC1* + *rbcL* | 553 | 63 |
| 2 | *matK* + *trnH-psbA* | 735 | 63 |
| 2 | *rbcL* + *trnH-psbA* | 592 | 64 |
| 2 | *matK* + *atpF-atpH* | 694 | 64 |
| 3 | *rpoC1* + *matK* + *trnH-psbA* | 868 | 65 |
| 3 | *trnH-psbA* + *atpF-atpH* + *psbK-psbI* | 921 | 66 |
| 4 | *rpoB* + *matK* + *trnH-psbA* + *atpF-atpH* | 1219 | 66 |
| 3 | *matK* + *trnH-psbA* + *atpF-atpH* | 1040 | 66 |
| 3 | *rpoB* + *rbcL* + *psbK-psbI* | 684 | 67 |
| 4 | *rpoB* + *rpoC1* + *matK* + *atpF-atpH* | 1010 | 67 |
| 5 | *rpoB* + *rpoC1* + *rbcL*+ *matK* + *trnH-psbA* | 1289 | 67 |
| 5 | *rpoC1* + *matK* + *trnH-psbA* + *atpF-atpH* + *psbK-psbI* | 1438 | 67 |
| 6 | *rpoB* + *rpoC1* + *matK* + *trnH-psbA* + *atpF-atpH* + *psbK-psbI* | 1617 | 67 |
| 4 | *matK* + *trnH-psbA* + *atpF-atpH* + *psbK-psbI* | 1302 | 67 |
| 4 | *rbcL* + *trnH-psbA* + *atpF-atpH* + *psbK-psbI* | 1163 | 68 |
| 4 | *rpoB* + *rpoC1* + *rbcL* + *psbK-psbI* | 816 | 68 |
| 4 | *rpoB* + *rpoC1* + *rbcL* + *matK* | 939 | 68 |
| 3 | *matK* + *atpF-atpH* + *psbK-psbI* | 956 | 69 |
| 5 | *rpoB* + *rpoC1* + *matK* + *atpF-atpH* + *psbK-psbI* | 1267 | 70 |
| 5 | *rpoB* + *rpoC1* + *rbcL*+ *matK* + *atpF-atpH* | 1243 | 70 |
| 5 | *rpoB* + *rbcL* + *trnH-psbA* + *atpF-atpH* + *psbK-psbI* | 1342 | 70 |
| 5 | *rpoB* + *rbcL*+ *matK* + *trnH-psbA* + *atpF-atpH* | 1376 | 70 |
| 4 | *rpoC1* + *rbcL* + *trnH-psbA* + *psbK-psbI* | 987 | 71 |
| 5 | *rpoC1* + *rbcL*+ *matK* + *trnH-psbA* + *psbK-psbI* | 1372 | 71 |
| 6 | *rpoB* + *rpoC1* + *rbcL*+ *matK* + *trnH-psbA* + *psbK-psbI* | 1551 | 71 |
| 6 | *rpoB* + *rpoC1* + *rbcL*+ *trnH-psbA* + *atpF-atpH* + *psbK-psbI* | 1474 | 71 |
| 6 | *rpoB* + *rpoC1* + *rbcL*+ *matK* + *atpF-atpH* + *psbK-psbI* | 1509 | 71 |
| 6 | *rpoC1* + *rbcL*+ *matK* + *trnH-psbA* + *atpF-atpH* + *psbK-psbI* | 1680 | 71 |
| 6 | *rpoB* + *rpoC1* + *rbcL*+ *matK* + *trnH-psbA* + *atpF-atpH* | 1590 | 71 |
| 6 | *rpoB* + *rbcL*+ *matK* + *trnH-psbA* + *atpF-atpH* + *psbK-psbI* | 1724 | 71 |
| 7 | *rpoB* + *rpoC1* + *rbcL*+ *matK* + *trnH-psbA* + *atpF-atpH* + *psbK-psbI* | 1859 | 71 |
