# Supplementary material for: Multiple Multilocus DNA Barcodes from the Plastid Genome Discriminate Plant Species Equally Well
Source: PLoS One. 2008 Jul 30;3(7):e2802. doi: 10.1371/journal.pone.0002802 (PMC2475660; doi:10.1371/journal.pone.0002802)
Supplement: Table S3 — GenBank and collection accession numbers for species sampled. (0.65 MB DOC) [file pone.0002802.s003.doc]

| OAC Accession No. | Family | Species | Genbank Accession No. | | | | | | | | |
| --- | --- | --- | --- | --- | --- | --- | --- | --- | --- | --- | --- |
|  |  |  | *cox1* | 23S rDNA | *rpoB* | *rpoC1* | *rbcL* | *matK* | *trnH-psbA* | *atpF-atpH* | *psbK-psbI* |
| OAC 88859 | Sapindaceae | *Acer negundo* | EU701103 | EU749953 | EU749049 | EU750189 | EU676886 | EU749280 | EU750427 | EU749500 | EU749739 |
| OAC 88939 | Sapindaceae | *Acer negundo* | EU701102 | EU749954 | EU749050 | EU750190 | EU676887 | EU749281 | EU750428 | EU749501 | EU749740 |
| OAC 88970 | Sapindaceae | *Acer platanoides* | EU701105 | EU749955 | EU749051 | EU750191 | EU676888 | EU749282 | EU750429 | EU749502 | EU749741 |
| OAC 89310 | Sapindaceae | *Acer platanoides* | EU701104 | EU749956 | EU749052 | EU750192 | EU676889 | EU749283 | EU750430 | EU749503 | EU749742 |
| OAC 88994 | Sapindaceae | *Acer rubrum* | EU701108 | EU749957 | EU749053 | EU750193 | EU676890 | EU749284 | EU750431 | EU749504 | EU749743 |
| OAC 89013 | Sapindaceae | *Acer rubrum* | EU701107 | EU749958 | EU749054 | EU750194 | EU676891 | EU749285 | EU750432 | EU749505 | EU749744 |
| OAC 89015 | Sapindaceae | *Acer rubrum* | EU701106 | EU749959 | EU749055 | EU750195 | EU676892 | EU749286 | EU750433 | EU749506 | EU749745 |
| OAC 88903 | Sapindaceae | *Acer saccharinum* | EU701111 | EU749960 | EU749056 | EU750196 | EU676893 | — | EU750434 | EU749507 | EU749746 |
| OAC 88940 | Sapindaceae | *Acer saccharinum* | EU701110 | EU749961 | EU749057 | EU750197 | EU676894 | EU749287 | EU750435 | EU749508 | EU749747 |
| OAC 89132 | Sapindaceae | *Acer saccharinum* | EU701101 | EU749962 | EU749058 | EU750198 | EU676895 | EU749288 | EU750436 | EU749509 | EU749748 |
| OAC 90015 | Sapindaceae | *Acer saccharinum* | EU701109 | EU749963 | EU749059 | EU750199 | EU676896 | EU749289 | EU750437 | EU749510 | EU749749 |
| OAC 88636 | Sapindaceae | *Acer saccharum* | EU701100 | EU749964 | EU749060 | EU750200 | EU676897 | EU749290 | EU750438 | EU749511 | EU749750 |
| OAC 88918 | Sapindaceae | *Acer saccharum* | EU701113 | EU749965 | EU749061 | EU750201 | EU676898 | EU749291 | EU750439 | EU749512 | EU749751 |
| OAC 88995 | Sapindaceae | *Acer saccharum* | EU701112 | EU749966 | EU749062 | EU750202 | EU676899 | EU749292 | EU750440 | EU749513 | EU749752 |
| OAC 88637 | Betulaceae | *Betula alleghaniensis* | EU701115 | EU749967 | EU749063 | EU750203 | EU676900 | EU749293 | EU750441 | — | EU749753 |
| OAC 89095 | Betulaceae | *Betula alleghaniensis* | EU701114 | EU749968 | EU749064 | EU750204 | EU676901 | EU749294 | EU750442 | — | EU749754 |
| OAC 88616 | Betulaceae | *Betula papyrifera* | EU701118 | EU749969 | EU749065 | EU750205 | EU676902 | EU749295 | EU750443 | — | EU749755 |
| OAC 88928 | Betulaceae | *Betula papyrifera* | EU701117 | EU749970 | EU749066 | EU750206 | EU676903 | EU749296 | EU750444 | — | EU749756 |
| OAC 89032 | Betulaceae | *Betula papyrifera* | EU701116 | EU749971 | EU749067 | EU750207 | EU676904 | EU749297 | EU750445 | — | EU749757 |
| OAC 88600 | Cornaceae | *Cornus alternifolia* | — | — | EU749068 | EU750208 | EU676905 | EU749298 | EU750446 | EU749514 | EU749758 |
| OAC 88937 | Cornaceae | *Cornus alternifolia* | EU701119 | EU749972 | EU749069 | EU750209 | EU676906 | EU749299 | EU750447 | EU749515 | EU749759 |
| OAC 88935 | Cornaceae | *Cornus racemosa* | EU701121 | EU749973 | EU749070 | EU750210 | EU676907 | EU749300 | EU750448 | EU749516 | EU749760 |
| OAC 89031 | Cornaceae | *Cornus racemosa* | EU701120 | EU749974 | EU749071 | EU750211 | EU676908 | EU749301 | EU750449 | EU749517 | EU749761 |
| OAC 88639 | Cornaceae | *Cornus rugosa* | EU701123 | EU749975 | EU749072 | EU750212 | EU676909 | EU749302 | EU750450 | EU749518 | EU749762 |
| OAC 88979 | Cornaceae | *Cornus rugosa* | EU701122 | EU749976 | EU749073 | EU750213 | EU676910 | EU749303 | EU750451 | EU749519 | EU749763 |
| OAC 88546 | Cornaceae | *Cornus stolonifera* | EU701125 | EU749977 | EU749074 | EU750214 | EU676911 | EU749304 | EU750452 | EU749520 | EU749764 |
| OAC 88896 | Cornaceae | *Cornus stolonifera* | EU701124 | EU749978 | EU749075 | EU750215 | EU676912 | EU749305 | EU750453 | EU749521 | EU749765 |
| OAC 88917 | Cornaceae | *Cornus stolonifera* | — | EU749979 | EU749076 | EU750216 | EU676913 | EU749306 | EU750454 | EU749522 | EU749766 |
| OAC 90009 | Cornaceae | *Cornus stolonifera* | — | EU749980 | EU749077 | EU750217 | EU676914 | EU749307 | EU750455 | EU749523 | EU749767 |
| OAC 88975 | Asteraceae | *Erigeron annuus* | EU701128 | EU749981 | EU749078 | EU750218 | EU676915 | EU749308 | EU750456 | EU749524 | EU749768 |
| OAC 88988 | Asteraceae | *Erigeron annuus* | EU701127 | EU749982 | EU749079 | EU750219 | EU676916 | EU749309 | EU750457 | EU749525 | EU749769 |
| OAC 89295 | Asteraceae | *Erigeron annuus* | EU701126 | EU749983 | EU749080 | EU750220 | EU676917 | EU749310 | EU750458 | EU749526 | EU749770 |
| OAC 88882 | Asteraceae | *Erigeron strigosus* | EU701132 | EU749984 | EU749081 | EU750221 | EU676918 | EU749311 | EU750459 | EU749527 | EU749771 |
| OAC 88897 | Asteraceae | *Erigeron strigosus* | EU701131 | EU749985 | EU749082 | EU750222 | EU676919 | EU749312 | EU750460 | EU749528 | EU749772 |
| OAC 88976 | Asteraceae | *Erigeron strigosus* | EU701130 | EU749986 | EU749083 | EU750223 | EU676920 | EU749313 | EU750461 | EU749529 | EU749773 |
| OAC 89089 | Asteraceae | *Erigeron strigosus* | EU701129 | EU749987 | EU749084 | EU750224 | EU676921 | EU749314 | EU750462 | EU749530 | EU749774 |
| OAC 88627 | Asteraceae | *Eupatorium maculatum* | EU701134 | EU749988 | EU749085 | EU750225 | EU676922 | EU749315 | EU750463 | EU749531 | EU749775 |
| OAC 89124 | Asteraceae | *Eupatorium maculatum* | EU701133 | EU749989 | EU749086 | EU750226 | EU676923 | EU749316 | EU750464 | EU749532 | EU749776 |
| OAC 88626 | Asteraceae | *Eupatorium perfoliatum* | EU701136 | EU749990 | EU749087 | EU750227 | EU676924 | EU749317 | EU750465 | EU749533 | EU749777 |
| OAC 89083 | Asteraceae | *Eupatorium perfoliatum* | EU701135 | EU749991 | EU749088 | EU750228 | EU676925 | EU749318 | EU750466 | EU749534 | EU749778 |
| OAC 90019 | Asteraceae | *Lactuca canadensis* | — | — | EU749089 | EU750229 | EU676926 | EU749319 | EU750467 | EU749535 | EU749779 |
| OAC 90023 | Asteraceae | *Lactuca canadensis* | — | — | EU749090 | EU750230 | EU676927 | EU749320 | EU750468 | EU749536 | EU749780 |
| OAC 89229 | Asteraceae | *Lactuca serriola* | EU701139 | EU749992 | EU749091 | EU750231 | EU676928 | EU749321 | EU750469 | EU749537 | EU749781 |
| OAC 90013 | Asteraceae | *Lactuca serriola* | — | — | EU749092 | EU750232 | EU676929 | EU749322 | EU750470 | EU749538 | EU749782 |
| OAC 88868 | Plantaginaceae | *Plantago lanceolata* | EU701154 | EU749993 | EU749093 | EU750233 | EU676930 | EU749323 | EU750471 | EU749539 | EU749783 |
| OAC 88980 | Plantaginaceae | *Plantago lanceolata* | EU701153 | EU749994 | EU749094 | EU750234 | EU676931 | EU749324 | EU750472 | EU749540 | EU749784 |
| OAC 88992 | Plantaginaceae | *Plantago lanceolata* | EU701152 | EU749995 | EU749095 | EU750235 | EU676932 | EU749325 | EU750473 | EU749541 | EU749785 |
| OAC 89007 | Plantaginaceae | *Plantago major* | EU701156 | EU749996 | EU749096 | EU750236 | EU676933 | EU749326 | EU750474 | EU749542 | EU749786 |
| OAC 89112 | Plantaginaceae | *Plantago major* | EU701155 | EU749997 | EU749097 | EU750237 | EU676934 | EU749327 | EU750475 | EU749543 | EU749787 |
| OAC 90020 | Plantaginaceae | *Plantago major* | — | EU749998 | EU749098 | EU750238 | EU676935 | EU749328 | EU750476 | EU749544 | EU749788 |
| OAC 89091 | Plantaginaceae | *Plantago rugelii* | EU701158 | EU749999 | EU749099 | EU750239 | EU676936 | EU749329 | EU750477 | EU749545 | EU749789 |
| OAC 89354 | Plantaginaceae | *Plantago rugelii* | EU701157 | EU750000 | EU749100 | EU750240 | EU676937 | EU749330 | EU750478 | EU749546 | EU749790 |
| OAC 88968 | Poaceae | *Poa annua* | EU701160 | EU750001 | EU749101 | EU750241 | EU676938 | EU749331 | EU750479 | EU749547 | EU749791 |
| OAC 89023 | Poaceae | *Poa annua* | EU701159 | EU750002 | EU749102 | EU750242 | EU676939 | EU749332 | EU750480 | EU749548 | EU749792 |
| OAC 88978 | Poaceae | *Poa compressa* | EU701163 | EU750003 | EU749103 | EU750243 | EU676940 | EU749333 | EU750481 | EU749549 | EU749793 |
| OAC 89109 | Poaceae | *Poa compressa* | EU701162 | EU750004 | EU749104 | EU750244 | EU676941 | EU749334 | EU750482 | EU749550 | EU749794 |
| OAC 89114 | Poaceae | *Poa compressa* | EU701161 | EU750005 | EU749105 | EU750245 | EU676942 | EU749335 | EU750483 | EU749551 | EU749795 |
| OAC 88632 | Polygonaceae | *Polygonum aviculare* | EU701167 | EU750006 | EU749106 | EU750246 | EU676943 | EU749336 | EU750484 | EU749552 | EU749796 |
| OAC 88926 | Polygonaceae | *Polygonum aviculare* | EU701166 | EU750007 | EU749107 | EU750247 | EU676944 | EU749337 | EU750485 | EU749553 | EU749797 |
| OAC 88974 | Polygonaceae | *Polygonum aviculare* | EU701165 | EU750008 | EU749108 | EU750248 | EU676945 | EU749338 | EU750486 | EU749554 | EU749798 |
| OAC 88989 | Polygonaceae | *Polygonum aviculare* | EU701164 | EU750009 | EU749109 | EU750249 | EU676946 | EU749339 | EU750487 | EU749555 | EU749799 |
| OAC 89090 | Polygonaceae | *Polygonum convolvulus* | EU701169 | EU750010 | EU749110 | EU750250 | EU676947 | EU749340 | EU750488 | EU749556 | EU749800 |
| OAC 89133 | Polygonaceae | *Polygonum convolvulus* | EU701168 | EU750011 | EU749111 | EU750251 | EU676948 | EU749341 | EU750489 | EU749557 | EU749801 |
| OAC 89067 | Polygonaceae | *Polygonum hydropiper* | EU701171 | EU750012 | EU749112 | EU750252 | EU676949 | EU749342 | EU750490 | EU749558 | EU749802 |
| OAC 89084 | Polygonaceae | *Polygonum hydropiper* | EU701170 | EU750013 | EU749113 | EU750253 | EU676950 | EU749343 | EU750491 | EU749559 | EU749803 |
| OAC 88962 | Polygonaceae | *Polygonum persicaria* | EU701174 | EU750014 | EU749114 | EU750254 | EU676951 | EU749344 | EU750492 | EU749560 | EU749804 |
| OAC 89088 | Polygonaceae | *Polygonum persicaria* | EU701173 | EU750015 | EU749115 | EU750255 | EU676952 | EU749345 | EU750493 | EU749561 | EU749805 |
| OAC 90002 | Polygonaceae | *Polygonum persicaria* | EU701172 | EU750016 | EU749116 | EU750256 | EU676953 | EU749346 | EU750494 | EU749562 | EU749806 |
| OAC 88930 | Salicaceae | *Populus balsamifera* | EU701176 | EU750017 | EU749117 | EU750257 | EU676954 | EU749347 | EU750495 | EU749563 | EU749807 |
| OAC 89065 | Salicaceae | *Populus balsamifera* | EU701175 | EU750018 | EU749118 | EU750258 | EU676955 | EU749348 | EU750496 | EU749564 | EU749808 |
| OAC 88942 | Salicaceae | *Populus deltoides* | EU701179 | EU750019 | EU749119 | EU750259 | EU676956 | EU749349 | EU750497 | EU749565 | EU749809 |
| OAC 89006 | Salicaceae | *Populus deltoides* | EU701178 | EU750020 | EU749120 | EU750260 | EU676957 | EU749350 | EU750498 | EU749566 | EU749810 |
| OAC 89087 | Salicaceae | *Populus deltoides* | EU701177 | EU750021 | EU749121 | EU750261 | EU676958 | EU749351 | EU750499 | EU749567 | EU749811 |
| OAC 88915 | Salicaceae | *Populus grandidentata* | EU701182 | EU750022 | EU749122 | EU750262 | EU676959 | EU749352 | EU750500 | EU749568 | EU749812 |
| OAC 88960 | Salicaceae | *Populus grandidentata* | EU701181 | EU750023 | EU749123 | EU750263 | EU676960 | EU749353 | EU750501 | EU749569 | EU749813 |
| OAC 89172 | Salicaceae | *Populus grandidentata* | EU701180 | EU750024 | EU749124 | EU750264 | EU676961 | EU749354 | EU750502 | EU749570 | EU749814 |
| OAC 88889 | Salicaceae | *Populus tremuloides* | EU701185 | EU750025 | EU749125 | EU750265 | EU676962 | EU749355 | EU750503 | EU749571 | EU749815 |
| OAC 88929 | Salicaceae | *Populus tremuloides* | EU701184 | EU750026 | EU749126 | EU750266 | EU676963 | EU749356 | EU750504 | EU749572 | EU749816 |
| OAC 90008 | Salicaceae | *Populus tremuloides* | EU701183 | EU750027 | EU749127 | EU750267 | EU676964 | EU749357 | EU750505 | EU749573 | EU749817 |
| OAC 88906 | Fagaceae | *Quercus alba* | EU701189 | EU750028 | EU749128 | EU750268 | EU676965 | — | EU750506 | EU749574 | EU749818 |
| OAC 88958 | Fagaceae | *Quercus alba* | EU701188 | EU750029 | EU749129 | EU750269 | EU676966 | EU749358 | EU750507 | EU749575 | EU749819 |
| OAC 88963 | Fagaceae | *Quercus alba* | EU701187 | EU750030 | EU749130 | EU750270 | EU676967 | EU749359 | EU750508 | EU749576 | EU749820 |
| OAC 89022 | Fagaceae | *Quercus alba* | EU701186 | EU750031 | EU749131 | EU750271 | EU676968 | EU749360 | EU750509 | EU749577 | EU749821 |
| OAC 90025 | Fagaceae | *Quercus cf.velutina* | EU701195 | EU750032 | EU749132 | EU750272 | EU676969 | EU749361 | EU750510 | EU749578 | EU749822 |
| OAC 88895 | Fagaceae | *Quercus macrocarpa* | EU701191 | EU750033 | EU749133 | EU750273 | EU676970 | EU749362 | EU750511 | EU749579 | EU749823 |
| OAC 88905 | Fagaceae | *Quercus macrocarpa* | EU701190 | EU750034 | EU749134 | EU750274 | EU676971 | EU749363 | EU750512 | EU749580 | EU749824 |
| OAC 88893 | Fagaceae | *Quercus rubra* | EU701194 | EU750035 | EU749135 | EU750275 | EU676972 | EU749364 | EU750513 | EU749581 | EU749825 |
| OAC 88993 | Fagaceae | *Quercus rubra* | EU701193 | EU750036 | EU749136 | EU750276 | EU676973 | EU749365 | EU750514 | EU749582 | EU749826 |
| OAC 90005 | Fagaceae | *Quercus rubra* | EU701192 | EU750037 | EU749137 | EU750277 | EU676974 | EU749366 | EU750515 | EU749583 | EU749827 |
| BPNP 267 | Rhamnaceae | *Rhamnus alnifolia* | — | — | EU749138 | EU750278 | EU676975 | EU749367 | EU750516 | EU749584 | EU749828 |
| OAC 77183 | Rhamnaceae | *Rhamnus alnifolia* | — | — | EU749139 | EU750279 | EU676976 | EU749368 | EU750517 | EU749585 | EU749829 |
| OAC 88924 | Rhamnaceae | *Rhamnus cathartica* | EU701198 | EU750038 | EU749140 | EU750280 | EU676977 | EU749369 | EU750518 | EU749586 | EU749830 |
| OAC 88967 | Rhamnaceae | *Rhamnus cathartica* | EU701197 | EU750039 | EU749141 | EU750281 | EU676978 | EU749370 | EU750519 | EU749587 | EU749831 |
| OAC 90004 | Rhamnaceae | *Rhamnus cathartica* | EU701196 | EU750040 | EU749142 | EU750282 | EU676979 | EU749371 | EU750520 | EU749588 | EU749832 |
| OAC 90016 | Rhamnaceae | *Rhamnus cathartica* | — | — | EU749143 | EU750283 | EU676980 | EU749372 | EU750521 | EU749589 | EU749833 |
| OAC 88922 | Rhamnaceae | *Rhamnus frangula* | EU701138 | EU750041 | EU749144 | EU750284 | EU676981 | EU749373 | EU750522 | EU749590 | EU749834 |
| OAC 88923 | Rhamnaceae | *Rhamnus frangula* | EU701137 | EU750042 | EU749145 | EU750285 | EU676982 | EU749374 | EU750523 | EU749591 | EU749835 |
| OAC 88871 | Rosaceae | *Rubus allegheniensis* | EU701201 | EU750043 | EU749146 | EU750286 | EU676983 | EU749375 | EU750524 | EU749592 | EU749836 |
| OAC 88982 | Rosaceae | *Rubus allegheniensis* | EU701200 | EU750044 | EU749147 | EU750287 | EU676984 | EU749376 | EU750525 | EU749593 | EU749837 |
| OAC 89016 | Rosaceae | *Rubus allegheniensis* | EU701199 | EU750045 | EU749148 | EU750288 | EU676985 | EU749377 | EU750526 | EU749594 | EU749838 |
| OAC 88886 | Rosaceae | *Rubus idaeus* | EU701206 | EU750046 | EU749149 | EU750289 | EU676986 | EU749378 | EU750527 | EU749595 | EU749839 |
| OAC 88891 | Rosaceae | *Rubus idaeus* | EU701205 | EU750047 | EU749150 | EU750290 | EU676987 | EU749379 | EU750528 | EU749596 | EU749840 |
| OAC 88961 | Rosaceae | *Rubus idaeus* | EU701204 | EU750048 | EU749151 | EU750291 | EU676988 | EU749380 | EU750529 | EU749597 | EU749841 |
| OAC 88983 | Rosaceae | *Rubus idaeus* | EU701203 | EU750049 | EU749152 | EU750292 | EU676989 | EU749381 | EU750530 | EU749598 | EU749842 |
| OAC 89011 | Rosaceae | *Rubus idaeus* | EU701202 | EU750050 | EU749153 | EU750293 | EU676990 | EU749382 | EU750531 | EU749599 | EU749843 |
| OAC 88705 | Rosaceae | *Rubus occidentalis* | EU701210 | EU750051 | EU749154 | EU750294 | EU676991 | EU749383 | EU750532 | EU749600 | EU749844 |
| OAC 88966 | Rosaceae | *Rubus occidentalis* | EU701209 | EU750052 | EU749155 | EU750295 | EU676992 | EU749384 | EU750533 | EU749601 | EU749845 |
| OAC 88987 | Rosaceae | *Rubus occidentalis* | EU701208 | EU750053 | EU749156 | EU750296 | EU676993 | EU749385 | EU750534 | EU749602 | EU749846 |
| OAC 89018 | Rosaceae | *Rubus occidentalis* | EU701207 | EU750054 | EU749157 | EU750297 | EU676994 | EU749386 | EU750535 | EU749603 | EU749847 |
| OAC 88931 | Rosaceae | *Rubus odoratus* | EU701212 | EU750055 | EU749158 | EU750298 | EU676995 | EU749387 | EU750536 | EU749604 | EU749848 |
| OAC 89183 | Rosaceae | *Rubus odoratus* | EU701211 | EU750056 | EU749159 | EU750299 | EU676996 | EU749388 | EU750537 | EU749605 | EU749849 |
| OAC 88934 | Salicaceae | *Salix* x*pendulina* | EU701219 | EU750057 | EU749160 | EU750300 | EU676997 | EU749389 | EU750538 | EU749606 | EU749850 |
| OAC 89309 | Salicaceae | *Salix* x*pendulina* | EU701220 | EU750058 | EU749161 | EU750301 | EU676998 | EU749390 | EU750539 | EU749607 | EU749851 |
| OAC 88879 | Salicaceae | *Salix eriocephala* | EU701216 | EU750059 | EU749162 | EU750302 | EU676999 | EU749391 | EU750540 | EU749608 | EU749852 |
| OAC 88900 | Salicaceae | *Salix eriocephala* | EU701215 | EU750060 | EU749163 | EU750303 | EU677000 | EU749392 | EU750541 | EU749609 | EU749853 |
| OAC 88941 | Salicaceae | *Salix eriocephala* | EU701214 | EU750061 | EU749164 | EU750304 | EU677001 | EU749393 | EU750542 | EU749610 | EU749854 |
| OAC 88969 | Salicaceae | *Salix eriocephala* | EU701213 | EU750062 | EU749165 | EU750305 | EU677002 | EU749394 | EU750543 | EU749611 | EU749855 |
| OAC 88904 | Salicaceae | *Salix exigua* | EU701218 | EU750063 | EU749166 | EU750306 | EU677003 | EU749395 | EU750544 | EU749612 | EU749856 |
| OAC 88977 | Salicaceae | *Salix exigua* | EU701217 | EU750064 | EU749167 | EU750307 | EU677004 | EU749396 | EU750545 | EU749613 | EU749857 |
| OAC 89075 | Caryophyllaceae | *Silene latifolia* | — | EU750065 | EU749168 | EU750308 | EU677005 | EU749397 | EU750546 | EU749614 | EU749858 |
| OAC 89093 | Caryophyllaceae | *Silene latifolia* | EU701221 | EU750066 | EU749169 | EU750309 | EU677006 | EU749398 | EU750547 | EU749615 | EU749859 |
| OAC 88876 | Caryophyllaceae | *Silene vulgaris* | — | EU750067 | EU749170 | EU750310 | EU677007 | EU749399 | EU750548 | EU749616 | EU749860 |
| OAC 88887 | Caryophyllaceae | *Silene vulgaris* | — | EU750068 | EU749171 | EU750311 | EU677008 | EU749400 | EU750549 | EU749617 | EU749861 |
| OAC 88953 | Caryophyllaceae | *Silene vulgaris* | — | EU750069 | EU749172 | EU750312 | EU677009 | EU749401 | EU750550 | EU749618 | EU749862 |
| OAC 88703 | Solanaceae | *Solanum dulcamara* | EU701224 | EU750070 | EU749173 | EU750313 | EU677010 | EU749402 | EU750551 | EU749619 | EU749863 |
| OAC 89008 | Solanaceae | *Solanum dulcamara* | EU701223 | EU750071 | EU749174 | EU750314 | EU677011 | EU749403 | EU750552 | EU749620 | EU749864 |
| OAC 89028 | Solanaceae | *Solanum dulcamara* | EU701222 | EU750072 | EU749175 | EU750315 | EU677012 | EU749404 | EU750553 | EU749621 | EU749865 |
| OAC 88965 | Solanaceae | *Solanum nigrum* | — | EU750073 | EU749176 | EU750316 | EU677013 | EU749405 | EU750554 | EU749622 | EU749866 |
| OAC 90003 | Solanaceae | *Solanum nigrum* | EU701226 | EU750074 | EU749177 | EU750317 | EU677014 | EU749406 | EU750555 | EU749623 | EU749867 |
| OAC 90026 | Solanaceae | *Solanum nigrum* | EU701225 | EU750075 | EU749178 | EU750318 | EU677015 | EU749407 | EU750556 | EU749624 | EU749868 |
| OAC 88907 | Asteraceae | *Solidago altissima* | EU701230 | EU750076 | EU749179 | EU750319 | EU677016 | EU749408 | EU750557 | EU749625 | EU749869 |
| OAC 89101 | Asteraceae | *Solidago altissima* | EU701229 | EU750077 | EU749180 | EU750320 | EU677017 | EU749409 | EU750558 | EU749626 | EU749870 |
| OAC 89113 | Asteraceae | *Solidago altissima* | EU701228 | EU750078 | EU749181 | EU750321 | EU677018 | EU749410 | EU750559 | EU749627 | EU749871 |
| OAC 89127 | Asteraceae | *Solidago altissima* | EU701227 | EU750079 | EU749182 | EU750322 | EU677019 | EU749411 | EU750560 | EU749628 | EU749872 |
| OAC 89030 | Asteraceae | *Solidago caesia* | EU701232 | EU750080 | EU749183 | EU750323 | EU677020 | EU749412 | EU750561 | EU749629 | EU749873 |
| OAC 89308 | Asteraceae | *Solidago caesia* | EU701231 | EU750081 | EU749184 | EU750324 | EU677021 | EU749413 | EU750562 | EU749630 | EU749874 |
| OAC 89094 | Asteraceae | *Solidago canadensis* | EU701234 | EU750082 | EU749185 | EU750325 | EU677022 | EU749414 | EU750563 | EU749631 | EU749875 |
| OAC 89317 | Asteraceae | *Solidago canadensis* | EU701233 | EU750083 | EU749186 | EU750326 | EU677023 | EU749415 | EU750564 | EU749632 | EU749876 |
| OAC 89020 | Asteraceae | *Solidago flexicaulis* | EU701236 | EU750084 | EU749187 | EU750327 | EU677024 | EU749416 | EU750565 | EU749633 | EU749877 |
| OAC 90006 | Asteraceae | *Solidago flexicaulis* | EU701235 | EU750085 | EU749188 | EU750328 | EU677025 | EU749417 | EU750566 | EU749634 | EU749878 |
| OAC 89105 | Asteraceae | *Solidago nemoralis* | EU701239 | EU750086 | EU749189 | EU750329 | EU677026 | EU749418 | EU750567 | EU749635 | EU749879 |
| OAC 89110 | Asteraceae | *Solidago nemoralis* | EU701238 | EU750087 | EU749190 | EU750330 | EU677027 | EU749419 | EU750568 | EU749636 | EU749880 |
| OAC 89130 | Asteraceae | *Solidago nemoralis* | EU701237 | EU750088 | EU749191 | EU750331 | EU677028 | EU749420 | EU750569 | EU749637 | EU749881 |
| OAC 88913 | Asteraceae | *Solidago rugosa* | EU701242 | EU750089 | EU749192 | EU750332 | EU677029 | EU749421 | EU750570 | EU749638 | EU749882 |
| OAC 88984 | Asteraceae | *Solidago rugosa* | EU701241 | EU750090 | EU749193 | EU750333 | EU677030 | EU749422 | EU750571 | EU749639 | EU749883 |
| OAC 89019 | Asteraceae | *Solidago rugosa* | EU701240 | EU750091 | EU749194 | EU750334 | EU677031 | EU749423 | EU750572 | EU749640 | EU749884 |
| OAC 89024 | Asteraceae | *Sonchus asper* | EU701245 | EU750092 | EU749195 | EU750335 | EU677032 | EU749424 | EU750573 | EU749641 | EU749885 |
| OAC 89027 | Asteraceae | *Sonchus asper* | EU701244 | EU750093 | EU749196 | EU750336 | EU677033 | EU749425 | EU750574 | EU749642 | EU749886 |
| OAC 89176 | Asteraceae | *Sonchus asper* | EU701243 | EU750094 | EU749197 | EU750337 | EU677034 | EU749426 | EU750575 | EU749643 | EU749887 |
| OAC 89025 | Asteraceae | *Sonchus oleraceus* | EU701246 | EU750095 | EU749198 | EU750338 | EU677035 | EU749427 | EU750576 | EU749644 | EU749888 |
| OAC 90014 | Asteraceae | *Sonchus oleraceus* | — | — | — | EU750339 | EU677036 | — | EU750577 | EU749645 | EU749889 |
| OAC 89100 | Asteraceae | *Symphyotrichum ciliolatum* | — | EU750096 | EU749199 | EU750340 | EU677037 | EU749428 | EU750578 | EU749646 | EU749890 |
| OAC 90012 | Asteraceae | *Symphyotrichum ciliolatum* | EU701247 | — | EU749200 | EU750341 | EU677038 | EU749429 | EU750579 | EU749647 | EU749891 |
| OAC 90027 | Asteraceae | *Symphyotrichum ciliolatum* | — | — | EU749201 | EU750342 | EU677039 | EU749430 | EU750580 | EU749648 | EU749892 |
| OAC 88925 | Asteraceae | *Symphyotrichum ericoides* | — | EU750097 | EU749202 | EU750343 | EU677040 | EU749431 | EU750581 | EU749649 | EU749893 |
| OAC 89111 | Asteraceae | *Symphyotrichum ericoides* | EU701248 | EU750098 | EU749203 | EU750344 | EU677041 | EU749432 | EU750582 | EU749650 | EU749894 |
| OAC 88919 | Asteraceae | *Symphyotrichum lanceolatum* | EU701250 | EU750099 | EU749204 | EU750345 | EU677042 | EU749433 | EU750583 | EU749651 | EU749895 |
| OAC 88921 | Asteraceae | *Symphyotrichum lanceolatum* | EU701249 | EU750100 | EU749205 | EU750346 | EU677043 | EU749434 | EU750584 | EU749652 | EU749896 |
| OAC 88861 | Asteraceae | *Symphyotrichum lateriflorum* | EU701251 | EU750101 | EU749206 | EU750347 | EU677044 | EU749435 | EU750585 | EU749653 | EU749897 |
| OAC 88973 | Asteraceae | *Symphyotrichum lateriflorum* | — | EU750102 | EU749207 | EU750348 | EU677045 | EU749436 | EU750586 | EU749654 | EU749898 |
| OAC 89017 | Asteraceae | *Symphyotrichum lateriflorum* | — | EU750103 | EU749208 | EU750349 | EU677046 | EU749437 | EU750587 | EU749655 | EU749899 |
| OAC 90022 | Asteraceae | *Symphyotrichum lateriflorum* | — | EU750104 | EU749209 | EU750350 | EU677047 | EU749438 | EU750588 | EU749656 | EU749900 |
| OAC 88892 | Asteraceae | *Symphyotrichum novae-angliae* | EU701252 | EU750105 | EU749210 | EU750351 | EU677048 | EU749439 | EU750589 | EU749657 | EU749901 |
| OAC 88899 | Asteraceae | *Symphyotrichum novae-angliae* | — | EU750106 | EU749211 | EU750352 | EU677049 | EU749440 | EU750590 | EU749658 | EU749902 |
| OAC 88927 | Asteraceae | *Symphyotrichum novae-angliae* | — | EU750107 | EU749212 | EU750353 | EU677050 | EU749441 | EU750591 | EU749659 | EU749903 |
| OAC 89170 | Asteraceae | *Symphyotrichum novae-angliae* | — | EU750108 | EU749213 | EU750354 | EU677051 | EU749442 | EU750592 | EU749660 | EU749904 |
| OAC 88914 | Asteraceae | *Symphyotrichum pilosum* | EU701254 | EU750109 | EU749214 | EU750355 | EU677052 | EU749443 | EU750593 | EU749661 | EU749905 |
| OAC 88936 | Asteraceae | *Symphyotrichum pilosum* | EU701253 | EU750110 | EU749215 | EU750356 | EU677053 | EU749444 | EU750594 | EU749662 | EU749906 |
| OAC 88938 | Asteraceae | *Symphyotrichum urophyllum* | EU701256 | EU750111 | EU749216 | EU750357 | EU677054 | EU749445 | EU750595 | EU749663 | EU749907 |
| OAC 89034 | Asteraceae | *Symphyotrichum urophyllum* | EU701255 | EU750112 | EU749217 | EU750358 | EU677055 | EU749446 | EU750596 | EU749664 | EU749908 |
| OAC 88687 | Fabaceae | *Trifolium pratense* | EU701259 | EU750113 | EU749218 | EU750359 | EU677056 | EU749447 | EU750597 | EU749665 | EU749909 |
| OAC 88910 | Fabaceae | *Trifolium pratense* | EU701258 | EU750114 | EU749219 | EU750360 | EU677057 | EU749448 | EU750598 | EU749666 | EU749910 |
| OAC 88954 | Fabaceae | *Trifolium pratense* | EU701257 | EU750115 | EU749220 | EU750361 | EU677058 | EU749449 | EU750599 | EU749667 | EU749911 |
| OAC 88688 | Fabaceae | *Trifolium repens* | EU701260 | EU750116 | EU749221 | EU750362 | EU677059 | EU749450 | EU750600 | EU749668 | EU749912 |
| OAC 88990 | Fabaceae | *Trifolium repens* | — | EU750117 | EU749222 | EU750363 | EU677060 | EU749451 | EU750601 | EU749669 | EU749913 |
| OAC 89026 | Fabaceae | *Trifolium repens* | — | EU750118 | EU749223 | EU750364 | EU677061 | EU749452 | EU750602 | EU749670 | EU749914 |
| OAC 89108 | Typhaceae | *Typha angustifolia* | EU701262 | EU750119 | EU749224 | EU750365 | EU677062 | EU749453 | EU750603 | EU749671 | EU749915 |
| OAC 90021 | Typhaceae | *Typha angustifolia* | EU701261 | EU750120 | EU749225 | EU750366 | EU677063 | EU749454 | EU750604 | EU749672 | EU749916 |
| OAC 88609 | Typhaceae | *Typha latifolia* | EU701264 | EU750121 | EU749226 | EU750367 | EU677064 | EU749455 | EU750605 | EU749673 | EU749917 |
| OAC 90024 | Typhaceae | *Typha latifolia* | EU701263 | EU750122 | EU749227 | EU750368 | EU677065 | EU749456 | EU750606 | EU749674 | EU749918 |
| OAC 88693 | Adoxaceae | *Viburnum acerifolium* | EU701266 | EU750123 | EU749228 | EU750369 | EU677066 | EU749457 | EU750607 | EU749675 | EU749919 |
| OAC 89085 | Adoxaceae | *Viburnum acerifolium* | EU701265 | EU750124 | EU749229 | EU750370 | EU677067 | EU749458 | EU750608 | EU749676 | EU749920 |
| OAC 89035 | Adoxaceae | *Viburnum lentago* | EU701268 | EU750125 | EU749230 | EU750371 | EU677068 | EU749459 | EU750609 | EU749677 | EU749921 |
| OAC 89228 | Adoxaceae | *Viburnum lentago* | EU701267 | EU750126 | EU749231 | EU750372 | EU677069 | EU749460 | EU750610 | EU749678 | EU749922 |
| OAC 88523 | Adoxaceae | *Viburnum opulus* | — | — | EU749232 | EU750373 | EU677070 | EU749461 | EU750611 | EU749679 | EU749923 |
| OAC 89029 | Adoxaceae | *Viburnum opulus* | EU701269 | EU750127 | EU749233 | EU750374 | EU677071 | EU749462 | EU750612 | EU749680 | EU749924 |
| OAC 88605 | Cupressaceae | *Juniperus communis* | — | EU750128 | EU749234 | EU750375 | EU677072 | EU749463 | EU750613 | EU749681 | EU749925 |
| OAC 88912 | Cupressaceae | *Juniperus communis* | — | EU750129 | EU749235 | EU750376 | EU677073 | EU749464 | EU750614 | EU749682 | EU749926 |
| OAC 89102 | Cupressaceae | *Juniperus communis* | — | EU750130 | EU749236 | EU750377 | EU677074 | EU749465 | EU750615 | EU749683 | EU749927 |
| OAC 90017 | Cupressaceae | *Juniperus communis* | — | EU750131 | EU749237 | EU750378 | EU677075 | EU749466 | EU750616 | EU749684 | — |
| OAC 88890 | Cupressaceae | *Juniperus virginiana* | — | EU750132 | EU749238 | EU750379 | EU677076 | EU749467 | EU750617 | EU749685 | EU749928 |
| OAC 88972 | Cupressaceae | *Juniperus virginiana* | — | EU750133 | EU749239 | EU750380 | EU677077 | EU749468 | EU750618 | EU749686 | EU749929 |
| OAC 89126 | Cupressaceae | *Juniperus virginiana* | — | EU750134 | EU749240 | EU750381 | EU677078 | EU749469 | EU750619 | EU749687 | — |
| OAC 89275 | Cupressaceae | *Juniperus virginiana* | — | EU750135 | EU749241 | EU750382 | EU677079 | EU749470 | EU750620 | EU749688 | — |
| OAC 88633 | Pinaceae | *Picea glauca* | — | — | EU749242 | — | EU677080 | — | EU750621 | EU749689 | EU749930 |
| OAC 89291 | Pinaceae | *Picea glauca* | EU701142 | EU750136 | EU749243 | EU750383 | EU677081 | EU749471 | EU750622 | EU749690 | EU749931 |
| OAC 89356 | Pinaceae | *Picea glauca* | EU701141 | EU750137 | EU749244 | EU750384 | EU677082 | EU749472 | EU750623 | EU749691 | EU749932 |
| OAC 90030 | Pinaceae | *Picea glauca* | EU701140 | EU750138 | EU749245 | EU750385 | EU677083 | EU749473 | EU750624 | EU749692 | EU749933 |
| OAC 89358 | Pinaceae | *Picea mariana* | — | — | EU749246 | EU750386 | EU677084 | EU749474 | EU750625 | EU749693 | EU749934 |
| OAC 90029 | Pinaceae | *Picea mariana* | EU701143 | EU750139 | EU749247 | — | EU677085 | EU749475 | EU750626 | EU749694 | EU749935 |
| OAC 89355 | Pinaceae | *Pinus banksiana* | EU701145 | EU750140 | EU749248 | EU750387 | EU677086 | EU749476 | EU750627 | EU749695 | — |
| OAC 89357 | Pinaceae | *Pinus banksiana* | EU701144 | EU750141 | EU749249 | EU750388 | EU677087 | EU749477 | EU750628 | EU749696 | EU749936 |
| OAC 89014 | Pinaceae | *Pinus strobus* | EU701148 | EU750142 | EU749250 | EU750389 | EU677088 | EU749478 | EU750629 | EU749697 | EU749937 |
| OAC 89096 | Pinaceae | *Pinus strobus* | EU701147 | EU750143 | EU749251 | EU750390 | EU677089 | EU749479 | EU750630 | EU749698 | EU749938 |
| OAC 90007 | Pinaceae | *Pinus strobus* | EU701146 | EU750144 | EU749252 | EU750391 | EU677090 | EU749480 | EU750631 | EU749699 | EU749939 |
| OAC 88959 | Pinaceae | *Pinus sylvestris* | EU701151 | EU750145 | EU749253 | EU750392 | EU677091 | EU749481 | EU750632 | EU749700 | EU749940 |
| OAC 88971 | Pinaceae | *Pinus sylvestris* | EU701150 | EU750146 | EU749254 | EU750393 | EU677092 | EU749482 | EU750633 | EU749701 | EU749941 |
| OAC 89125 | Pinaceae | *Pinus sylvestris* | EU701149 | EU750147 | EU749255 | EU750394 | EU677093 | EU749483 | EU750634 | EU749702 | EU749942 |
| OAC 88880 | Dryopteridaceae | *Dryopteris carthusiana* | — | EU750148 | — | — | EU677094 | — | EU750635 | EU749703 | EU749943 |
| OAC 88955 | Dryopteridaceae | *Dryopteris carthusiana* | — | EU750149 | — | EU750395 | EU677095 | — | EU750636 | EU749704 | EU749944 |
| OAC 89353 | Dryopteridaceae | *Dryopteris carthusiana* | — | EU750150 | — | EU750396 | EU677096 | — | EU750637 | EU749705 | EU749945 |
| OAC 88916 | Dryopteridaceae | *Dryopteris intermedia* | — | EU750151 | — | EU750397 | EU677097 | — | EU750638 | EU749706 | EU749946 |
| OAC 88991 | Dryopteridaceae | *Dryopteris intermedia* | — | EU750152 | — | EU750398 | EU677098 | — | — | EU749707 | EU749947 |
| OAC 89012 | Dryopteridaceae | *Dryopteris intermedia* | — | EU750153 | — | EU750399 | EU677099 | — | EU750639 | EU749708 | EU749948 |
| OAC 90011 | Dryopteridaceae | *Dryopteris intermedia* | — | EU750154 | — | EU750400 | EU677100 | — | EU750640 | EU749709 | EU749949 |
| OAC 88986 | Dryopteridaceae | *Dryopteris marginalis* | — | EU750155 | — | EU750401 | EU677101 | — | EU750641 | EU749710 | EU749950 |
| OAC 89021 | Dryopteridaceae | *Dryopteris marginalis* | — | EU750156 | — | EU750402 | EU677102 | — | EU750642 | EU749711 | EU749951 |
| OAC 90010 | Dryopteridaceae | *Dryopteris marginalis* | — | EU750157 | — | EU750403 | EU677103 | — | EU750643 | EU749712 | — |
| OAC 90018 | Dryopteridaceae | *Dryopteris marginalis* | — | EU750158 | — | EU750404 | EU677104 | — | EU750644 | EU749713 | EU749952 |
| OAC 88908 | Equisetaceae | *Equisetum arvense* | — | EU750159 | — | — | EU677105 | — | EU750645 | — | — |
| OAC 89128 | Equisetaceae | *Equisetum arvense* | — | EU750160 | — | — | EU677106 | — | EU750646 | — | — |
| OAC 88964 | Equisetaceae | *Equisetum hyemale* | — | EU750161 | — | — | EU677107 | EU749484 | — | — | — |
| OAC 88981 | Equisetaceae | *Equisetum hyemale* | — | EU750162 | — | — | EU677108 | EU749485 | EU750647 | — | — |
| OAC 89000 | Equisetaceae | *Equisetum hyemale* | — | EU750163 | — | — | EU677109 | EU749486 | EU750648 | — | — |
| OAC 89141 | Equisetaceae | *Equisetum hyemale* | — | EU750164 | — | — | EU677110 | EU749487 | EU750649 | — | — |
| OAC 88573 | Lycopodiaceae | *Lycopodium digitatum* | — | EU750165 | EU749256 | EU750405 | EU677111 | EU749488 | EU750650 | EU749714 | — |
| OAC 88920 | Lycopodiaceae | *Lycopodium digitatum* | — | EU750166 | EU749257 | EU750406 | EU677112 | EU749489 | EU750651 | EU749715 | — |
| OAC 89033 | Lycopodiaceae | *Lycopodium obscurum* | — | EU750167 | EU749258 | — | EU677113 | EU749490 | EU750652 | EU749716 | — |
| OAC 89086 | Lycopodiaceae | *Lycopodium obscurum* | — | EU750168 | EU749259 | — | EU677114 | EU749491 | EU750653 | EU749717 | — |
| OAC 89134 | Lycopodiaceae | *Lycopodium obscurum* | — | EU750169 | EU749260 | — | EU677115 | EU749492 | EU750654 | EU749718 | — |
| OAC 90028 | Lycopodiaceae | *Lycopodium obscurum* | — | EU750170 | — | — | EU677116 | — | EU750655 | EU749719 | — |
| OAC 91225 | Brachytheciaceae | *Brachythecium oxycladon* | — | EU750171 | EU749261 | EU750407 | EU677117 | — | EU750656 | EU749720 | — |
| OAC 91226 | Brachytheciaceae | *Brachythecium oxycladon* | — | EU750172 | EU749262 | EU750408 | EU677118 | EU749493 | EU750657 | EU749721 | — |
| OAC 91227 | Brachytheciaceae | *Brachythecium reflexum* | — | EU750173 | EU749263 | EU750409 | EU677119 | EU749494 | EU750658 | EU749722 | — |
| OAC 91228 | Brachytheciaceae | *Brachythecium reflexum* | — | EU750174 | EU749264 | EU750410 | EU677120 | EU749495 | EU750659 | EU749723 | — |
| OAC 91229 | Brachytheciaceae | *Brachythecium salebrosum* | — | EU750175 | EU749265 | EU750411 | EU677121 | — | EU750660 | EU749724 | — |
| OAC 91230 | Brachytheciaceae | *Brachythecium salebrosum* | — | EU750176 | EU749266 | EU750412 | EU677122 | EU749496 | EU750661 | EU749725 | — |
| OAC 91231 | Dicranaceae | *Dicranum flagellare* | — | EU750177 | EU749267 | EU750413 | EU677123 | — | EU750662 | EU749726 | — |
| OAC 91232 | Dicranaceae | *Dicranum flagellare* | — | EU750178 | EU749268 | EU750414 | EU677124 | — | EU750663 | EU749727 | — |
| OAC 91233 | Dicranaceae | *Dicranum flagellare* | — | EU750179 | EU749269 | EU750415 | EU677125 | — | EU750664 | — | — |
| OAC 91234 | Dicranaceae | *Dicranum polysetum* | — | EU750180 | EU749270 | EU750416 | EU677126 | — | EU750665 | EU749728 | — |
| OAC 91235 | Dicranaceae | *Dicranum polysetum* | — | — | EU749271 | EU750417 | EU677127 | — | EU750666 | EU749729 | — |
| OAC 91236 | Mniaceae | *Plagiomnium cuspidatum* | — | EU750181 | EU749272 | EU750418 | EU677128 | EU749497 | EU750667 | EU749730 | — |
| OAC 91237 | Mniaceae | *Plagiomnium cuspidatum* | — | EU750182 | EU749273 | EU750419 | EU677129 | EU749498 | EU750668 | EU749731 | — |
| OAC 91238 | Mniaceae | *Plagiomnium drummondii* | — | EU750183 | EU749274 | EU750420 | EU677130 | EU749499 | EU750669 | EU749732 | — |
| OAC 91239 | Mniaceae | *Plagiomnium drummondii* | — | — | — | EU750421 | EU677131 | — | EU750670 | EU749733 | — |
| OAC 91240 | Polytrichaceae | *Polytrichum commune* | — | EU750184 | EU749275 | EU750422 | EU677132 | — | EU750671 | EU749734 | — |
| OAC 91241 | Polytrichaceae | *Polytrichum commune* | — | EU750185 | EU749276 | EU750423 | EU677133 | — | EU750672 | EU749735 | — |
| OAC 91242 | Polytrichaceae | *Polytrichum juniperinum* | — | EU750186 | EU749277 | EU750424 | EU677134 | — | EU750673 | EU749736 | — |
| OAC 91243 | Polytrichaceae | *Polytrichum juniperinum* | — | EU750187 | EU749278 | EU750425 | EU677135 | — | EU750674 | EU749737 | — |
| OAC 91244 | Polytrichaceae | *Polytrichum juniperinum* | — | EU750188 | EU749279 | EU750426 | EU677136 | — | EU750675 | EU749738 | — |
